# Supplementary material for: Microtubule stiffening by the doublecortin-domain protein ZYG-8 contributes to mitotic spindle orientation during zygote division in Caenorhabditis elegans
Source: PLoS Genet. 2026 Jun 29;22(6):e1012196. doi: 10.1371/journal.pgen.1012196 (PMC13340844; doi:10.1371/journal.pgen.1012196)
Supplement: Attachment — Submitted filename: Cueff_2026_Response_to_reviewers.pdf [file pgen.1012196.s037.pdf]

Point-by-point for the article “**Microtubule stiffening by the doublecortin-domain protein ZYG-8 contributes to mitotic spindle orientation during zygote division in *Caenorhabditis elegans.***”

Reviewer #1:

Summary
This manuscript by Cueff et al investigates how the doublecortin domain protein ZYG 8 regulates spindle positioning and oscillations in the C. elegans zygote. Using three perturbation strategies, (i) a temperature sensitive (ts) mutant affecting the first doublecortin domain, (ii) overexpression by additional expression of ZYG-8 from the pie 1 promoter, and (iii) RNAi depletion, the authors show that ZYG-8 localizes to the mitotic spindle, including astral microtubules, and that its loss increases the oscillation amplitude while decreasing frequency. They further analyze microtubule (MT) growth and nucleation via EBP-2 tracking and centrosome size, examine cortical MT contact dynamics, and propose that ZYG-8 modulates MT flexural rigidity, thereby altering the balance of pushing and pulling forces on the spindle. The topic is significant, and the dataset is rich, but the manuscript is dense and sometimes confusing, and several comparisons (e.g., SPD-2, CLS-2) are not the most informative. The rationale for analyzing cortical MT contacts and DiLiPop separately is unclear, as both address MT number and lifetime at the cortex, with the only difference that in the DiLiPop assay the authors classify short MT lifetimes at the cortex as pulling and long life times as pushing events. Overall, the work is interesting and relevant but requires some clarification.

Major Strengths
Multiple independent perturbations (CRISPR ts allele, overexpression, RNAi) increase confidence that phenotypes are due to ZYG-8.
Clear primary phenotype: ZYG-8 loss increases oscillation amplitude and reduces frequency; ts allele shows stronger effect than RNAi, consistent with reduced MT binding.
Integration of approaches: imaging, quantitative analysis, and modeling.
Novel mechanistic model linking doublecortin-dependent MT rigidity to spindle force balance.

We sincerely thank the Reviewer #1 for her/his thoughtful and encouraging assessment of our study. We are particularly pleased that she/he highlighted these key strengths, which are central to our experimental design and work.

Major Concerns

1) The logic excluding growth and nucleation effects is hard to follow because comparisons are not equivalent in magnitude or mechanism.

We thank the Reviewer for raising this concern regarding the clarity of our logical framework for excluding microtubule growth and nucleation effects. We set out to design these experiments with *zyg-9(RNAi)* and *spd-2(RNAi)* to achieve effects of comparable magnitude to those observed in *zyg-8* mutants (for microtubule growth) and *zyg-8* overexpression (for microtubule nucleation). The detailed clarifications provided below address this point, and we hope they will resolve the reviewer’s concern.

As an example, the ZYG 8 ts reduces the MT growth rate by ~15%, to exclude that this reduction in growth rate is what affects the oscillation, the authors choose RNAi of the MT polymerase ZYG-9. Depletion of ZYG 9 was reported to reduce MT growth rates by ~3 fold and to shorten spindles. Even though the authors use partial RNAi, which presumably has less effect on MT growth rates, those conditions might not be comparable and variable.

We agree with the reviewer that Srayko *et al.* (2005) reported a ~3-fold reduction (–72%) in microtubule growth rate upon ZYG-9 depletion via injection. To avoid this extreme effect, we performed a partial ZYG-9 depletion using a 6-hour RNAi feeding protocol and quantified the growth rate reduction relative to controls. Under these conditions, the growth rate decreased by 16%, closely matching the 15% reduction observed in *zyg-8* mutants.

To clarify a potentially misleading passage in the main text p. 7, we have revised it as follows (changes shown in bold):

"To test whether this 15% reduction in microtubule growth could account for the exaggerated spindle-pole oscillations observed in *zyg-8* mutants, we performed a partial RNAi depletion of the microtubule polymerase ZYG-9^{XMAP215} [47, 84, 85]. **This treatment shortened the mitotic spindle (Figure S4A), confirming efficient depletion [84], and reduced the microtubule growth rate by 16%, comparable to the effect in *zyg-8* mutants (Figure S4B).**"

In addition, the authors find that ZYG 8 depletion increases MT nucleation, to exclude an effect of increased MT nucleation on oscillations the authors choose to compare ZYG-8 depletion to SPD-2 depletion. However, SPD 2 depletion decreases MT nucleation, which is the opposite as reported for ZYG-8. Thus, it is not a good control to test the effect on oscillations of an increased MT nucleation. The authors should consider instead a perturbation that elevates nucleation, i.e. KLP-7 (however there are caveats with KLP-7 as well, see below).

Our comparison with SPD-2 depletion was not intended to mimic ZYG-8 depletion (which increases nucleation) but rather to parallel *zyg-8* overexpression, as both conditions reduce microtubule nucleation (Figure 3C,G and S5B). We acknowledge that our original wording may have been unclear, and we have revised the main text, p. 7, as follows to better reflect this rationale (changes shown in bold):

"Next, we tested whether altered nucleation alone could explain the spindle-pole oscillation phenotype observed upon *zyg-8* perturbation. …/… ***spd-2(RNAi)* caused a 21% reduction in centrosome diameter (Figure S5A, B), an effect comparable to—but slightly more pronounced than—that of *zyg-8* overexpression (Figure 3E, G). While *zyg-8* overexpression increases oscillation frequency by 20% (Figure 1D), *spd-2(RNAi)* decreased frequency by 11% (Figure S5D). Neither condition significantly altered oscillation amplitude (Figures 1C and S5C).**"

We recognize that KLP-7 depletion would indeed increase nucleation and could serve as a complementary control. However, we opted against this approach because our focus was on isolating nucleation defects without confounding effects, particularly the altered microtubule stability due to KLP-7's role as a depolymerase.

The section comparing ZYG 8 to CLASP (CLS 2) and KLP 7 is difficult to interpret because both of these perturbations strongly affect midzone integrity and frequently cause spindle rupture, unlike ZYG 8 manipulations. In addition, both KLP-7 and CLS-2 are mainly acting on the chromosomes to correct errors and promote kinetochore MT growth, respectively. Since both KLP-7 and CLS-2, alter global spindle architecture and do not just affect global MT polymerization, they are not very good mechanistic controls for astral MT behavior. If the aim is to control for polymerase activity, ZYG 9 (carefully titrated) would be the more appropriate control.

We agree with the Reviewer that KLP-7 and CLS-2 perturbations affect the mitotic spindle, particularly midzone integrity and kinetochore-microtubule interactions. However, published evidence also demonstrates that these proteins regulate microtubule dynamics in the cytoplasm and at the cell cortex (Espiritu, *et al*., 2012; Bouvrais *et al*., 2021). For this reason, we used these perturbations to compare their phenotypes with those obtained when we targeted *zyg-8*. For *klp-7(RNAi)*, we employed a hypomorphic treatment (24-hour RNAi feeding) that altered microtubule dynamics without inducing spindle break, as evidenced by spindle elongation (Fig. S7A). This suggests that midzone integrity was largely preserved upon our *klp-7(RNAi)* treatment. For *cls-2(RNAi)*, we performed a 48-hour RNAi feeding to target cortical microtubule dynamics. This led to faster spindle elongation (Fig. S6A), indicating some midzone perturbation; we acknowledge this as a limitation. Nonetheless, we believe these experiments remain informative, particularly since Reviewer #2 requested additional data to show the phenotypes of cortical microtubule dynamics upon these perturbations, which we have now provided. In a broader take, as suggested by the reviewer, it is not possible to target a single feature of microtubule dynamics; we thus performed a panel of hypomorphic depletions to be interpreted collectively to state that ZYG-8 effect on microtubule dynamics is not enough to account for its phenotype (see last paragraph of section 2 of the results).
We have revised the text (p. 8) as follows to better reflect how these two depletions affect or not spindle midzone integrity (addition shown in bold):

"We validated the penetrance of the RNAi treatment by observing accelerated mitotic spindle elongation, **which suggested midzone integrity perturbations** [97, 98] (Figure S6A)."

"We confirmed *klp-7(RNAi)* treatment penetrance **by observing slightly increased spindle elongation (suggesting only a mild alteration of midzone integrity)** and longer metaphase spindle, as expected [99] (Figure S7A)."

These additional works, requested by Reviewer #2, demonstrate that *klp-7(RNAi)* and *cls-2(RNAi)* affect cortical microtubule count or dynamics, consistent with their known roles. The partial depletion of the depolymerase KLP-7 increased the number of microtubules reaching the cell cortex and prolonged cortical lifetimes (Figure S7F, G), aligning with its destabilizing role. Depletion of CLS-2—a proposed catastrophe "reducer" and/or stabilizing factor—decreased cortical microtubule count and shortened cortical lifetimes during anaphase (Figure S6F, G), consistent with its stabilizing role.
Further details on these experiments, including associated figure and text revisions, are provided in our response to Reviewer #2 (p. 15-16).

Overall, while those comparisons are used by the authors to emphasize that changes in MT dynamics alone do not cause the same effects on oscillations, I am not sure they are helpful in

supporting this message. I think these comparisons could be moved to Supplement and it would be helpful to point out the limitations of each of them in the discussion.

We appreciate the reviewer's perspective on the comparative analyses of *zyg-9(RNAi)*, *spd-2(RNAi)*, *csl-2(RNAi)* and *klp-7(RNAi)* experiments. While we understand the concern that these comparisons may not fully isolate microtubule dynamics effects, we believe they remain valuable in the main text, particularly as Reviewer #2 requested their inclusion. To address the reviewer's suggestion, we propose to (1) retain these comparisons in the main text with reinforced clarifications and additional data, and (2) add a dedicated paragraph in the discussion to explicitly discuss the limitations of these experimental approaches.

We have provided the following text for the Discussion (p. 15):

"While our comparisons using depletions of various MAP provide valuable insights into the role of microtubule dynamics in spindle oscillations and cortical microtubule contact phenotypes, we acknowledge some limitations. First, some perturbations introduced additional confounding effects, e.g. *cls-2(RNAi)* affecting midzone integrity, complicating direct comparisons with *zyg-8* perturbations. Second, cumulative effects of multiple perturbations may contribute to the phenotypes observed upon *zyg-8* targeting. Nonetheless, taken collectively, our set of experiments suggests that alterations in microtubule dynamics alone cannot fully recapitulate the spindle-pole oscillation variations, spindle final position defects, and cortical microtubule contact phenotypes observed upon *zyg-8* targeting. This supports our conclusion that ZYG-8 plays an additional role beyond simply regulating microtubule dynamics."

2) The rationale for analyzing cortical MT contacts and DiLiPop separately is unclear since both assess MT number and lifetime at the cortex. The mayor difference seems to be that depending on the lifetime (short vs long) the microtubules are counted as pulling or pushing.

We appreciate the reviewer's question regarding our two-step analysis of cortical microtubule contacts. Below, we clarify the rationale for this approach and propose to incorporate this explanation into the Materials and Methods (M10) section.
The two analyses serve distinct purposes and address different biological questions at separate stages of our study:
(1) The initial quantification, previoulsy employed in the literature (O'Rourke *et al*., 2010; Espiritu *et al*., 2012), establishes the basic parameters of cortical microtubule behavior, excluding microtubule dynamics as the sole cause of ZYG-8 alteration phenotype.
(2) The DiLiPop modeling, introduced in Bouvrais *et al.* (2021), provides mechanistic insights into force generation to offer an additional role for this protein, i.e. reinforce microtubule rigidity.

We have clarified this in the Materials and Methods (M10) section with the following amended text p. 24 (addition shown in bold):

"After tracking the microtubule contacts, **we analysed them using a two-step approach to examine distinct aspects of microtubule-cortical interactions**.
(1) <u>Basic quantification</u>. We quantified the number of microtubule cortical contacts per frame, averaged them over a 10-second window, and normalised them by the embryo area. …/…

This analysis assessed whether ZYG-8 contributes to microtubule stabilisation by preventing e.g. cytoplasmic depolymerisation or cortical catastrophe events.

(2) **DiLiPop (Distinct Lifetime Population) analysis.** This statistical analysis of the microtubule cortical contact durations reveals the short-lived and long-lived populations of astral microtubules at the cortex associated with pulling and pushing events, respectively [80]. **This analysis evaluated ZYG-8's role in cortical force generation**."

Based on the DiLiPop assay the authors discard an effect of pulling, which seems a bit unclear. While they detect longer lifetimes, which the authors argue are indicative of more pushing they also state that the measured reduction in density of pulling events (short time) could be due to prolonged duration of individual pulling. This would however mean that some of the longer time events could then actually be pulling events and not pushing events and would thus be falsely classified by the assay. Maybe the authors could clarify this.

We acknowledge that our current presentation of the DiLiPop assay results may not fully clarify why we conclude that ZYG-8 does not significantly affect pulling forces. To address this, we propose to supplement our analysis with additional DiLiPop experiments comparing *zyg-8(RNAi)* to perturbations known to specifically alter pulling forces.
To test whether the increased spindle-pole oscillation amplitudes in *zyg-8(RNAi)* embryos could result from enhanced cortical pulling forces, we performed two key comparisons.
We compared the *zyg-8(RNAi)* DiLiPop phenotype with that of *gbp-1(RNAi)*, as GBP-1 depletion is known to increase cortical pulling forces (Afshar et al., 2004; Afshar et al., 2010; Thyagarajan *et al*., 2011). While *gbp-1(RNAi)* did not significantly alter the lifetimes of pulling or pushing events, it caused a significant increase in pulling event density and a decrease in pushing event density This phenotype differs markedly from that observed in *zyg-8(RNAi)* embryos and is consistent with an increase in pulling force through greater pulling event density. Conversely, when we reduced cortical pulling forces via *gpr-1/2(RNAi);gpr-2(0)*, we observed a decrease in pulling event density, an increase in pushing event density, and no change in event lifetimes. This phenotype is the opposite of what we observed in *gbp-1(RNAi)* and further supports our interpretation.
We have complemented Figure 7B-E (showing DiLiPop results for *zyg-8(RNAi)*) with new panels (Figure 7F-I) presenting the *gbp-1(RNAi)* data. This direct comparison will strengthen our conclusion that cortical pulling forces are not significantly increased in *zyg-8(RNAi)* embryos.
We have amended the relevant text as follows p. 11 (addition shown in bold):

"However, these modest changes in pulling events could not account for the strong increase in oscillation amplitudes. **For comparison, when we depleted GBP-1 to enhance cortical pulling force [111-113], we observed a significant increase in pulling-event density, as expected, while the lifetimes of both pulling and pushing events remained unaffected (Figure 7F-I). This phenotype differed from that of *zyg-8(RNAi)*, leading us to conclude that *zyg-8(RNAi)* had at most a minor effect on cortical pulling forces**."

Regarding the second point addressed by the reviewer, our previous statement - "*Since all cortical force generators are engaged in pulling during anaphase, this reduced density likely reflected the prolonged duration of individual pulling events*" – may have been misleading. To clarify:
Under the assumption that the total number of force generators remains constant between *zyg-8(RNAi)* and control embryos, and knowing that all force generators are actively pulling during

anaphase, we reason as follows: Since we measure the number of events (i.e. the number of microtubule contact tracks at the cortex and not the instantaneous density of these contacts), if individual pulling events last longer (increased lifetime), then fewer distinct pulling events can occur within the same time window (reduced density).
This explains the simultaneous observation of increased pulling-event lifetimes and decreased pulling-event density in *zyg-8(RNAi)* embryos.
We have amended the relevant text as follows p. 11 (addition shown in bold):

"Upon *zyg-8(RNAi)*, we observed a 10% rise in the lifetime of the pulling events (Figure 7B) and a 3% reduction in their density (Figure 7C). **Since these measurements reflect the number of events (i.e., the number of microtubule cortical tracks, not the instantaneous density of cortical contacts), longer-lasting pulling events (increased lifetimes) reduce the number of distinct pulling events that can occur within the same time window (reduced density). This occurs because all available** cortical force generators are engaged in pulling during anaphase [66, 72, 75], **explaining the simultaneous observation of increased pulling-event lifetimes and decreased pulling-event density in *zyg-8(RNAi)* embryos**."

3) Evidence for pulling forces being "largely unaffected" relies on indirect metrics (centrosome position, DiLiPop) and is partially contradictory.
The authors could consider laser ablation to directly test pulling forces, or acknowledge limitations.

We acknowledge that our current analyses (centrosome positioning and DiLiPop) do not constitute direct measurements of cortical pulling forces. The Reviewer suggests using laser ablation as a more direct approach. However, we note that laser ablation also represents an indirect method, as its readouts reflect the net balance of both pulling and pushing forces acting on the spindle. In contrast, our DiLiPop analysis offers the advantage of discriminating between cortical pulling and pushing events based on their distinct lifetime signatures.
That said, we agree that additional experimental evidence would strengthen our conclusions. Following a suggestion from Reviewer #2, we have now quantified cortical dynein counts and dynamics. These new data are presented in our response to Reviewer #2 (p. 13).

Along this line, On Page 19: posterior centrosome position is used to argue that there is no change in pulling forces as the position is comparable to wildtyp after zyg-8 (RNAi), in contrast page 22 states that in the ZYG 8 ts, the centrosome is closer to the cortex. Please reconcile this difference.

We appreciate the reviewer's careful attention to the apparent discrepancy regarding centrosome position measurements. We would like to clarify that these represent two distinct measurements.
To assess potential changes in cortical pulling forces, we specifically measured the most posterior position of the posterior centrosome projected along the anteroposterior axis during anaphase in *zyg-8(RNAi)* embryos. This focused measurement revealed no significant difference compared to controls. We acknowledge that this projection methodology was not clearly stated in the original text. Thus, we have clarified it as follows p. 11 (addition shown in bold):

"To distinguish between these possibilities, we first measured the most posterior position of the posterior centrosome, **projected along the anteroposterior (AP) axis**, during anaphase following *zyg-8(RNAi)* treatment. We found no significant difference compared to controls (80.7 ± 1.4 % **of the AP axis** for *zyg-8(RNAi)* ($N = 16$), versus 79.8 ± 1.0 % for control RNAi embryos ($N = 10$), Student's *t*-test: $p = 0.07$))."

The second readout referred to, namely the closer proximity to the cortex, i.e. the distance between the centrosome and the cortex in any direction, and it differs fundamentally from the first readout, as it does not project the position along the AP axis and combines both position and orientation in 3D space. Importantly, in *zyg-8(ts)* mutants, spindle orientation defects during anaphase prevent using the most-posterior centrosome position as a reliable readout of cortical pulling force.

Cytosim simulations are a strength, but parameter choices (rigidity values from in vitro data) should be justified and ideally linked to measured curvature distributions.

As noted by the reviewer, we based our selection of microtubule rigidity values on published *in vitro* measurements (ranging from 2 to 25 pN·µm²) due to the current lack of *in vivo* rigidity data. We fully acknowledge that (1) the presence of microtubule-associated proteins *in vivo* could modify these values, (2) *C. elegans*-specific tubulin might exhibit different mechanical properties, and (3) the cellular environment may influence microtubule rigidity beyond what is captured *in vitro*.
However, the key insight from our simulations does not depend on the absolute rigidity values. Rather, it relies on observing how variations in rigidity within this plausible range affect microtubule behavior. Specifically, we demonstrate an anti-correlation between microtubule rigidity and cortical lifetime. This qualitative relationship is what supports our biological conclusions, not the precise quantitative values of rigidity.

To clarify this in the manuscript, we have added the following text to the Material & Method section (M12) p. 27 (addition shown in bold):

"Their rigidities were set to 2, 10, or 25 pN·µm², reflecting the range *of in vitro* measurements, varying between 3.7 and 26 pN. µm² [21], **due to the current lack of *in vivo* rigidity values**. **While the actual rigidity in *C. elegans* zygotes may differ due to the presence of MAPs, tubulin specificity, and cellular environment factors, we aimed at linking relative rigidity variations and microtubule cortical residency to support our conclusions.**"

4) The figures could benefit from more clear organization and consistency. Along this line, the figures sometimes show only RNAi or only ts mutant, not both. This can appear selective.

We appreciate the reviewer's feedback regarding figure organisation and consistency. We acknowledge that some figures do not include all the experimental conditions, which may create an impression of selective data presentation.
Where feasible, we have added the missing experimental conditions to the relevant figures to ensure comprehensive representation of our data. In cases where certain conditions were not included, we have provided clear explanations in the figure legends to justify these omissions (e.g., technical limitations, or irrelevance to the specific point being illustrated).

Below, we address the reviewer's specific concerns in greater detail.

As an example, Figure 4 F shows the relative contact count for zyg-8 RNAi for contacts between 0.2-0.3s and 0.3-0.4s, the data the reader should compare this to is in Figure 4H. However, Figure 4H shows the relative count for zyg-8 overexpression and only for events longer than 0.4s, this makes it difficult to compare as the two plots show essentially data for different groups of MT contacts.

We appreciate the reviewer's careful observation regarding the inconsistent presentation of data in Figure 4F and 4H. We acknowledge that the previous organisation made direct comparisons challenging. To address this issue and improve comparability, we have modified panel F by focussing on the proportion of contacts with durations below or equal to 0.4 s.

Data for the ZYG-8 ts strain is not shown at all in Figure 4, while it appeared in previous figures.

As previously mentioned, we were unable to accurately quantify cortical microtubule contacts in *zyg-8* mutants due to severe spindle misorientation during anaphase. High fluorescence for the centrosome prevented reliable data collection for this specific analysis. We recognize that including these data would have provided valuable comparative insights.

To clarify this, we have added the following text to the legend of Figure 4:
"This figure does not include data for *zyg-8(or484ts)* mutant, as severe spindle misorientation precluded reliable quantification for this analysis."

Similarly Figure 5 only shows data for ZYG-8 ts and control but not for zyg-8 (RNAi) or overexpression. Figure 5 could also be combined with Figure 6.

To address the concern about missing conditions, we have completed the analysis by examining astral microtubule curvature phenotypes of live embryos during anaphase across all relevant conditions by including *zyg-8(RNAi)* treatment and *zyg-8* overexpression.
While we recognize the value of presenting all conditions in a single figure, we chose to distribute these data across multiple figures to maintain visual clarity by avoiding overcrowded figures and to ensure each panel remains large enough for detailed observation. We have created two new supplemental figures (Figures S9 and S10) showing the additional conditions and provided associated supplemental movies (Movies S8-S11) from which the images were extracted. We have added a statement to the legends of Figures 5, S9 and S10 to clarify that these figures display comparable features across the various conditions targeting *zyg-8*. For example, the legend of Figure 5 now includes the following note:
"Similar images for *zyg-8(RNAi)* and *zyg-8* overexpression are shown in Figures S9 and S10, with associated movies provided as Movies S8-S11."

We have supplemented the main text as follows p. 9 to consider these new experiments (addition shown in bold):

"We first asked whether astral microtubules displayed increased local curvature in *zyg-8(or484ts)* mutants **or in *zyg-8(RNAi)*-treated embryos**. …/… **Consistently, astral**

microtubules exhibited increased bending in live *zyg-8(RNAi)*-treated embryos (Figure S9A, Movie S8) compared to control RNAi embryos (Figure S9B, Movie S9). In contrast, *zyg-8* overexpression in live embryos did not significantly alter microtubule curvature amplitudes or frequencies (Figure S10, Movies S10 & S11). …/... ”

In Figure 6, we omitted data for *zyg-8* overexpressing embryos, as live imaging (Figure S10) revealed no detectable changes in astral microtubule curvatures compared to untreated embryos.
To clarify this, we have added the following statement to the legend of Figure 6:
“This figure does not include data for *zyg-8* overexpressing embryos, as live imaging (Figure S10) demonstrated no significant differences in the astral microtubule curvatures.”

In Figure 6 A and B the data shows the curvature for ZYG-8 (RNAi) but then C and D show the proportion of MTs with a certain curvature for the ZYG-8 ts mutant.

The current presentation may have led to some confusion. To clarify, all four panels (A-D) in Figure 6 do indeed show the proportion of microtubules across curvature bins (ranging from 0 to 1 μm^{-1}). Panels A1 and B1 display the full curvature distribution for *zyg-8(RNAi)* and control conditions, respectively. Panels C and D show the same analysis for *zyg-8* mutants and their controls.
We have improved this figure by adding focused insets (C2, D2) that highlight the proportion of microtubules in the lowest curvature range (below 0.1 μm^{-1}) for *zyg-8* mutants and their controls, similar to what we showed for *zyg-8(RNAi)* in the current A2 and B2 panels.

In Figure 7 only data for the DiLiPop assay in zyg-8 (RNAi) is shown, no data for the ZYG-8 ts or overexpression.
Figure 8 shows only data for the TS mutant.
The use of different conditions in different figures should either be justified, i.e. why does Figure 7 only show data for zyg-8 (RNAi) and Figure 8 only the TS mutant, or the different conditions should be consistently included in main figures.

For Figure 7, we were unable to perform the DiLiPop assay in *zyg-8* mutants due to severe spindle mispositioning during anaphase, which prevented reliable quantification of cortical microtubule contacts. We acknowledge that these data would have provided valuable comparative insights. *zyg-8* overexpression was intentionally omitted from Figure 7 because this section of the manuscript specifically focuses on mechanistic explanations for increased spindle-pole oscillation amplitudes, which are only present in *zyg-8(RNAi)*-treated embryos and in *zyg-8* mutants.
To clarify these omissions, we have added the following statement to the legend of Figure 7:
“This figure does not include data for *zyg-8(or484ts)* mutant, as severe spindle misorientation precluded reliable quantification. Additionally, data for *zyg-8* overexpressing embryos are not included, as this figure focuses on elucidating the origin of increased spindle-pole oscillation amplitudes—a phenotype not observed in this condition (Figure 1C).”

We have complemented Figure 8 with data from *zyg-8(RNAi)*-treated embryos (panels E, F), which revealed no major defects in spindle positioning and orientation at anaphase end. This addition strengthens our conclusion in Section 5 ("*Very soft microtubules disrupt final spindle positioning and orientation*") by demonstrating that only excessively soft microtubules (as in

zyg-8 mutants) lead to positioning defects. Since this section focuses specifically on the consequences of excessively soft microtubules, we determined that including *zyg-8* overexpression condition would not contribute meaningfully to the core message of this figure. To clarify this, we have added the following statement to the legend of Figure 8:
"Data for *zyg-8* overexpressing embryos are not included in this figure, as it focuses on the consequences of reduced microtubule rigidity—a phenotype not observed under this condition (Figure S10)."

Typo: Fig. 7B, in the text it says the figure shows pushing but based on the figure I believe it should be pulling.

We thank the reviewer for catching this typo. We confirm that this was indeed an error. We have corrected the text to accurately reflect that Figure 7B represents pulling event characteristics.

In summary, the core findings are interesting and potentially impactful. Addressing the comparability of controls, clarifying analyses, and resolving text inconsistencies will substantially improve the manuscript.

We sincerely appreciate the reviewer's thoughtful and constructive feedback, which has been invaluable in strengthening our manuscript. We have carefully addressed each of the reviewer's concerns. We believe these revisions have substantially improved the clarity, rigor, and presentation of our work, and have resulted in a significantly strengthened manuscript.

Reviewer #2:

The manuscript by Cueff et al. focuses on the role of ZYG8/DCX in spindle orientation in C. elegans. The manuscript's strength lies in the number and variety of microscopy-based quantifications performed on fluorescently labeled microtubule data from both live and fixed samples. The authors identify spindle phenotypes associated with ZYG8 perturbations and explore multiple avenues to elucidate the underlying mechanism explaining these phenotypes. Authors demonstrate that ZYG8 perturbations result in a reduction in microtubule growth rate (Figure 2), an increase in microtubule nucleation (Figure 3), and an increase in cortical contacts (Figure 4). Can these subcellular microtubule-associated phenotypes explain the spindle oscillation phenotype? To answer this question, the authors compare the effect of ZYG8 perturbations on spindle oscillations with that of other genes involved in regulating microtubule growth rate (XMAP), nucleation (CEP192), stabilization (CLASP), destabilization (MCAK), or stiffness (TAU). All these explorations lead the authors to propose that the role of ZYG8 in spindle orientation and oscillations stems not from its involvement in microtubule dynamics, but rather from its effect on microtubule stiffness. I found that the quantification and analysis of ZYG8 phenotypes were well done and convincing; however, the argument regarding microtubule rigidity as the unique causal parameter was somewhat weaker. Indeed, the microtubule shape analysis used to infer microtubule stiffness is based on an untested assumption, and the comparisons between ZYG8 and other microtubule regulators are incomplete, as microtubule properties (dynamics, rigidity) are not systematically analyzed in the conditions where known microtubule regulators are perturbed, only spindle phenotypes are.

Overall, the strengths of the manuscript provide a solid foundation on which the authors can further refine their mechanistic conclusions. Below are some suggestions that I hope will be helpful in strengthening the manuscript's main points.

We deeply thank the Reviewer #2 for this evaluation of our manuscript. We greatly appreciate the recognition of our study's strengths as well as the constructive criticism, which has helped us identifiy areas of improvement.
We indeed recognize that ZYG-8 plays a role in both microtubule rigidity regulation and microtubule dynamics. Importantly, our results suggest that ZYG-8's mechanical contribution to the observed phenotypes is more significant than its dynamic effects.

The main text has been clarified on this point in several places. A few examples are quoted below:
<u>In the result section</u>:
(p. 14): "Together, these observations further supported our conclusion that reduced microtubule rigidity, rather than altered microtubule dynamics, is the determining factor of spindle misorientation in *zyg-8(or484ts)* mutants."
<u>In the discussion section</u>:
(p. 15): "Nonetheless, taken collectively, our set of experiments suggests that alterations in microtubule dynamics alone cannot fully recapitulate the spindle-pole oscillation variations, spindle final position defects, and cortical microtubule contact phenotypes observed upon *zyg-8* targeting. This supports our conclusion that ZYG-8 plays an additional role beyond simply regulating microtubule dynamics."
(p. 16): "Our data suggest that, while ZYG-8 contributes to microtubule dynamics, its primary role in regulating spindle positioning and orientation is mediated through modulation of microtubule rigidity, particularly under conditions of elevated cortical pulling forces (Figure 8A-D). This mechanical function likely represents the dominant mechanism accounting for the observed phenotypic effects, with alterations in microtubule dynamics playing a secondary role."

We believe the revisions proposed below will substantially strengthen our manuscript by providing a more nuanced and well-supported mechanistic explanation for ZYG-8's role in spindle orientation.

<u>Major comments</u>:

• Measuring microtubule stiffness or rigidity is very difficult, if not impossible, in cells, as recognized by the authors at the beginning of section 3 of the results. The authors should be commended for tackling an inherently challenging question and for being transparent about the limitations. The authors decide to rely on a shape analysis to deduce potential changes in rigidity. This is a very classical comparison often made in mechanics classes: if two beams are subjected to the same load, but one bends more than the other, then their mechanical properties must be different. The converse is equally true: if two beams have different shapes under load but have the same mechanical properties, the loads must be different. One difference is that in the first case, where identical loads but different mechanical properties are involved, the external forces are the same for both beams—they push back on the load with the same overall force— even though they bend by different amounts.
If we let the beams in our examples be microtubules, the thought experiment remains valid. However, there are now two unknown parameters, the load and the mechanical properties,

which prevent reaching a conclusion just from the shape analysis alone. Again, the authors recognize that limitation and decide to work “under the assumption that the forces acting on the microtubule network are similar between conditions.” This assumption is critical, as it determines how strongly the conclusions can be supported. Because the assumption is so critical to the reasoning, and because authors are making rigidity a big part of their story, it is crucial to test the validity of this assumption. I appreciate that the authors are thinking carefully about force-generating mechanisms as demonstrated by the initial analysis provided. The astral microtubules can experience force from three different sources: dynein motors anchored to the cortex, dynein motors moving cargo along the microtubules, and cortical pushing when the microtubules grow against the cell cortex. The authors must estimate how each of these contributors to forces is affected by the perturbations in ZYG8 to determine if the assumption is valid. I recognize that they partially do it by measuring the number and duration of the cortical contacts. However, while these data begin to address the question, additional information would clarify the robustness the assumption.
The amount of dynein could change, the amount of dynein engaged at the cortex, or on organelles could change, the distribution of organelles could change, the amount of dynein per microtubule could change, etc... The effect of ZYG8 perturbations on the amount of force originating from dynein activity and localization needs to be looked at to properly assess the validity of the assumption and, consequently this part of the reasoning. If additional supporting data are not available, it may help to moderate the strength of the conclusion about rigidity. Critically, a paragraph in section 4 of the results seems to suggest that astral microtubules in CTRL and ZYG8 backgrounds are not experiencing the same force: “We interpreted the decrease in fc as primarily reflecting a centering force reduction due to softer microtubules.” which would indicate that the assumption is not true.

We thank the reviewer for raising this concern regarding our rigidity measurements and the underlying assumptions. We fully recognize the importance of this issue and have taken several steps to address it in our revision.

In our approach, demonstrating that the cortical pulling forces are not substantially increased upon *zyg-8(RNAi)* or in *zyg-8* mutants is key, since it is indeed a possible mechanism that could explain microtubule curvature change. To investigate this possibility, we have performed two additional experiments.

(1) In response to Reviewer #1's comment, we added a comparison of DiLiPop signatures from the landing assay (M10) between *zyg-8(RNAi)* and *gpb-1(RNAi)* conditions. Given that GPB-1 depletion is established to increase cortical pulling forces in *C. elegans* zygotes (Afshar et al., 2004; Afshar et al., 2010; Thyagarajan *et al.*, 2011), the distinct DiLiPop profiles observed between these conditions provide additional evidence against a substantial increase in pulling forces upon *zyg-8(RNAi)* (Figure 7F-I). Specifically, *gpb-1(RNAi)* resulted in a significant increase in the density of the short-lived population (Figure 7G), consistent with enhanced cortical pulling forces, while the lifetime of this population remained unchanged (Figure 7F). In contrast, *zyg-8(RNAi)* led to a slight decrease in short-lived population density and a modest increase in its lifetime (Figure 7B, C). These divergent profiles highlight the distinct mechanical consequences of *zyg-8* and *gpb-1* depletion.
We have updated the main text to present the DiLiPop analysis results for the *gpb-1(RNAi)* condition and its corresponding control, as explained in our answer to Reviewer #1 (p. 5).

(2) To further test this hypothesis, we have conducted new experiments tracking cortical dynein in *zyg-8(RNAi)* and control RNAi backgrounds. However, we note that changes in dynein count

at the cortex may not directly translate to variations in cortical pulling forces. This is because cortical force generators are trimeric complexes comprising GPR-1/2^{LGN}, LIN-5^{NuMA} and dynein, and GPR-1/2 are thought to be the limiting factor for complex assembly at the cell cortex. Indeed, in (Rodriguez-Garcia et al., 2018), they demonstrated that the limiting factor in pulling force generation was not the cortical presence of dynein, but its engagement (k_{on}) in generating such forces. Besides, several studies proposed that cortical force-generators are saturated during anaphase (Grill et al., 2005; Pécréaux et al., 2006; Bouvrais et al., 2018).
These new data are presented in the Section 4 of the Results and Table S3, alongside its Method as M13. Our analysis revealed two distinct dynamic behaviours of the cortical dyneins in both the control and *zyg-8(RNAi)* embryos.

	**Control RNAi (*N* = 11)**		***zyg-8(RNAi)* (*N* = 14)**	
	Population #1	Population #2	Population #1	Population #2
**Lifetime (s)**	0.66	1.66	0.74	2.07
**Proportion (%)**	86	14	84	16
**Density (/min/µm²)**	0.79	0.13	0.95	0.18

The short-lived population (~0.7 s) likely includes the cortical pulling events, since it matches the lifetime of microtubules involved in pulling events (Bouvrais et al., 2021), while the role of the long-lived population, which is minoritar (14-16% of the tracks), would benefit from additional investigation beyond the scope of this study. Indeed, dyneins are involved in multiple molecular pathways in *C. elegans* zygotes (Gusnowski et al., 2011; Boudreau et al., 2019) and in other systems (e.g., Gutierrez et al., 2017). For instance, this long-lived population may reflect pushing events, as dynein depletion impaired both pulling and pushing leading to mispositionned spindle (Gonczy et al., 1999).
Upon *zyg-8(RNAi)*, we observed an increase in both the lifetimes and densities of the two populations, which aligns with the increase in cortical microtubule contact density and lifetime (Figure 4C, E). Since the magnitude of these changes is comparable for dyneins and microtubules, this suggests that ZYG-8 does not directly regulate dynein activity or cortical pulling forces. Furthermore, an increase in dynein density does not necessarily result in increased cortical pulling force, as only dyneins actively engaged with cortical force generators (including GPR-1/2) contribute to pulling (Schmidt et al., 2017; Rodriguez-Garcia et al., 2018). Given that cortical GPR-1/2 availability is the limiting factor—and these proteins are saturated during anaphase (Bouvrais et al., 2018)—no major increase in cortical pulling force is expected, even though cortical dynein density increases upon *zyg-8(RNAi)*.

The additional text in the Results section reads as follows p. 12:
"To further investigate whether cortical pulling forces remain unaffected when targeting *zyg-8*, we tracked cortical dynein using a CRISPR strain expressing GFP::DHC-1 [114] (Method M13), a biophysical assay previously employed [114, 115]. It is important to note that only cortical dynein molecules incorporated into at least the GPR-1/2-LIN-5-dynein trimeric complex actively generate pulling forces on astral microtubules [74, 116]. In control RNAi embryos, we identified two distinct dynein populations at the cell cortex: a short-lived population with a lifetime of 0.66 s, and a long-lived population with a lifetime of 1.66 s (Table S3). The lifetime of the predominant short-lived population aligns with previously reported measurements [115], and falls within the range of cortical pulling event lifetimes (Figure 7B, F), suggesting that this population likely includes active pulling events. Upon *zyg-8(RNAi)*, our analysis revealed similar dynein dynamics (Table S3). The increased dynein density aligns with the elevated microtubule contact density (Figure

4C), while the prolonged dynein lifetimes mirror the increased microtubule cortical contact durations. Crucially, the magnitude of these changes in dynein dynamics was comparable to those observed for microtubule characteristics, suggesting no additional regulatory mechanism. Given that not all cortical dynein is engaged in pulling [114, 115] and that cortical force generators are saturated (i.e., actively pulling) during anaphase in untreated embryos [66, 72, 75], it is unlikely that ZYG-8 depletion substantially increases cortical pulling forces."

It is well established that the dominant forces during anaphase are pulling. Thus, the key drivers of microtubule curvature generation *in cellulo* are pulling forces. We rather suggest that the pushing forces, indeed decreased upon depleting ZYG-8, can be viewed as a secondary readout, revealing the decreased forces upon microtubule buckling under load, rather than the cause of curvature.
We clarified this by adding the word "pulling" and the revised text now reads (p. 9):

"Such an approach has been previously used as a proxy for rigidity under the assumption that the **pulling** forces acting on the microtubule network are similar between conditions [20, 104, 105]."

Last, we have complemented the discussion that now explicitly addresses the mechanical interpretation of our curvature data (p. 15):

"Given that Doublecortin family proteins have been proposed to regulate microtubule rigidity, we investigated whether ZYG-8 performs a similar function in *C. elegans* zygotes. …/… **Increased cortical pulling forces upon zyg-8(RNAi) could be thought to alternatively explain the observed increase in microtubule bending. However, such a view contradicts our DiLiPop experiments (Figure 7B, C). Additionally, cortical dynein tracking revealed that changes in dynein behaviour were insufficient to account for a large increase in pulling forces (Table S3). While we did observe increased dynein density—consistent with more microtubules reaching the cortex upon ZYG-8 depletion—this did not translate into increased pulling events, as GPR-1/2 association in the force-generating complex and dynein engagements remain the limiting factors. The increase in cortical dynein lifetimes aligned with the prolonged residency times of cortical microtubules, without suggesting an additional contribution. We conclude that ZYG-8 primarily acts by increasing microtubule rigidity rather than by regulating cortical pulling force generation**."

• When authors discuss the dynamics of cortical microtubules (section 2 of the Results), they first describe the number of contacts and their duration in various ZYG8 perturbations. The idea here is, on the one hand, to understand if ZYG8 is a microtubule stabilizer (like other DCX family members) and if that role could explain the phenotypes observed on the spindle. The data indicate that ZYG8 is not a stabilizer in their hands but the opposite. Then, to try to tease out whether the destabilizing role of ZYG8 on microtubules can explain the spindle phenotypes, the authors compare ZYG8 conditions to those of two mutants, CLASP and MCAK. That comparison remains superficial, as the authors only examined spindle orientation in these mutants. Extending the authors' thoughtful comparison to include additional microtubule-level analyses would further strengthen their argument. To fully understand the picture, authors should analyze the dynamics of cortical microtubules and microtubule shape distribution in these mutants in the same way they did for the ZYG8 conditions (number of contacts and duration, tortuosity, and curvature). It would first validate that CLASP and MCAK

are indeed acting as stabilizers and destabilizers, respectively, and importantly, that their analysis pipeline can detect it, supporting the ZYG8 analysis. Notably, since the CLASP mutant exhibits some spindle phenotypes (albeit different from those of ZYG8), it would be interesting to understand if the dynamics of cortical microtubules are altered or if microtubule shape profile is changed in this background, and whether this can explain the observed spindle phenotypes.

We appreciate the Reviewer #2's valuable suggestion to include more comprehensive microtubule-level analyses for *klp-7*^{MCAK}*(RNAi)* and *cls-2*^{CLASP}*(RNAi)* conditions. In response, we have expanded our analyses to systematically characterize cortical microtubule dynamics in these genetic backgrounds, using the same quantitative approaches applied to *zyg-8* perturbations.
Later in the manuscript, we aimed to demonstrate that spindle final misorientation and mispositioning in *zyg-8* mutants are primary due to microtubule rigidity impairement. We therefore investigated whether depletion of CLS-2 or KLP-7 would recapitulate the spindle misorientation and mispositioning phenotypes observed in *zyg-8* mutants. Our findings revealed no significant changes in spindle final position or orientation in either *cls-2(RNAi)* or *klp-7(RNAi)* embryos (Figure S6D,E; Figure S7D,E) This suggests that alterations in microtubule catastrophe or depolymerization alone are insufficient to induce spindle misorientation in *C. elegans* zygotes. Given these results, we determined that performing extensive microtubule curvature quantification in *cls-2(RNAi)* and *klp-7(RNAi)* backgrounds—which would require substantial additional work—would not be essential for addressing our central hypothesis regarding ZYG-8's mechanical role.

Our cortical microtubule dynamics analysis of *cls-2(RNAi)* embryos revealed reduced microtubule contact counts during anaphase (Figure S6F) and decreased proportions of long-duration contacts (Figure S6G). These findings align perfectly with CLS-2's established role as a microtubule stabiliser that prevents catastrophe, particularly under conditions of elevated cortical pulling forces. The observed phenotypes serve as an important positive control, validating our analytical pipeline's ability to detect microtubule stabilisation effects.
In *klp-7(RNAi)* embryos, we observed increased microtubule contact counts (Figure S7F) and higher proportions of long-duration contacts during anaphase (Figure S7G). These results are fully consistent with KLP-7's known function in promoting microtubule depolymerization.
Crucially, when comparing these cortical microtubule signatures with those observed in *zyg-8(RNAi)* embryos, we find patterns that do not support a primary role for ZYG-8 in microtubule stabilisation. This comparison strengthens our conclusion that ZYG-8 plays an additional role beyond conventional microtubule dynamics regulation.
To accommodate these new analyses, we have split the original Supplementary Figure S6 into two separate figures: Figure S6 that focused exclusively on *cls-2(RNAi)* data and Figure S7 dedicated to *klp-7(RNAi)* results. This reorganization improves clarity and allows for more detailed presentation of each condition.

Additionally, we have supplemented the main text p. 8 as follows (addition shown in bold):

"To further test this finding, we compared cortical microtubule dynamics and spindle pole oscillations in *zyg-8(RNAi)*-treated embryos with those following depletion of CLS-2^{CLASP} …/… **At the cell cortex, in *cls-2(RNAi)*-treated embryos, we found a reduced density of microtubule contacts during anaphase (Figure S6F), and a decreased proportion of long-duration contacts (Figure S6G). These results aligned with CLS-2's known role in catastrophe prevention, but differed from *zyg-8(RNAi)* phenotypes, further suggesting**

that ZYG-8 does not primarily prevent microtubule catastrophe in *C. elegans* zygote. …/…
We then compared *zyg-8(RNAi)* phenotypes with those following hypomorphic depletion of KLP-7^{MCAK}, …/… **At the cell cortex, KLP-7 depletion increased the microtubule contact density and the proportion of long-duration contacts (Figure S7F, G). These findings reflected impaired depolymerisation activity. If ZYG-8 stabilised microtubules by preventing depolymerisation, we would have observed cortical microtubule phenotypes opposite to that of KLP-7 depletion. This was not the case.** …/…"

We have also supplemented the discussion p. 14 with these additional data as follows (modifications shown in bold):

"We first confirmed previous findings by Srayko *et al.*, …/… If ZYG-8's main function were to stabilise microtubules by reducing their catastrophe rate at the cell cortex, *zyg-8(RNAi)* treatment would be expected to decrease the cortical lifetimes of astral microtubules **and reduce their contact counts. This phenotype would resemble what we observed with *cls-2(RNAi)* (Figure S6F, G). In contrast, we found the opposite effect: *zyg-8(RNAi)* increases both astral microtubule contact counts and durations (Figure 4C, E).** Upon *klp-7(RNAi)*, which reduces microtubule depolymerisation, **we found an increase in both microtubule contact counts and durations (Figure S7F, G). If ZYG-8 primarily acted to prevent microtubule depolymerization, the phenotypes would have differed markedly from those in *zyg-8(RNAi)*—yet they remained similar."**

• To assess if microtubule rigidity can explain the phenotypes observed authors decided to explore the phenotype associated with PTL-1 depletion as a positive control. I value and support this attempt, but it seems incomplete on two fronts. Firstly, the authors only looked at spindle orientation phenotypes. Comparing the cortical microtubule dynamics and microtubule shape associated with PTL-1 RNAi with those of ZYG8 perturbations would be a more effective way to assess the similarities in mechanism between the two conditions.

We thank the reviewer for raising this point regarding PTL-1. We acknowledge that investigating cortical microtubule dynamics and microtubule curvatures upon *ptl-1(RNAi)* could provide valuable insights.
Our analysis revealed that changes in spindle pole oscillations upon *ptl-1(RNAi)* are substantially weaker than those observed with *zyg-8(RNAi)*. Given that detecting significant microtubule shape changes in *zyg-8(RNAi)* embryos was already challenging, we anticipate that *ptl-1(RNAi)* would likely show no statistically significant alterations in microtubule curvature. Consequently, we have chosen not to include this time-consuming experiment in our current revision.
In response to the reviewer's concern, we have moderated our conclusions regarding PTL-1 by removing references to PTL-1 from the Results (Section 4), the end of the Introduction, and the Abstract, and by retaining references to PTL-1 in the Discussion section (see below) to provide context for future studies.

Secondly, another microtubule rigidity regulator exists and appears to be expressed in C. elegans, SPD-1, which belongs to the Ase1/PRC1/MAP65-1 family. Exploring the spindle orientation phenotypes and cortical microtubule dynamics phenotypes in SPD-1 depletion seems important.

We appreciate the reviewer's suggestion to examine SPD-1, a member of the Ase1/PRC1/MAP65-1 family known to regulate microtubule rigidity in other systems. While SPD-1 has been shown to decrease flexural rigidity of individual microtubules and increase microtubule-bundle flexibility (Portran *et al*., 2013), its role in *C. elegans* zygotes remains unclear. Specifically:

- SPD-1 localizes to the spindle midzone during anaphase (Verbrugghe *et al*., 2004 (Fig. S2A-C); Wallis *et al*., 2015 (Fig. 2B); Zimyanin *et al*., 2023 (Fig. 3D)).
- No evidence exists for its localization on astral microtubules.
- Previous studies using anti-tubulin staining or GFP::tubulin labeling in *spd-1(oj5)* mutants showed no major changes in microtubule shapes (Verbrugghe *et al*., 2004 (Fig. S2I-K); Santos *et al*., 2023 (Fig. 3)).

Despite these limitations, we investigated spindle pole oscillations upon *spd-1(RNAi)*; we confirmed treatment efficacy through accelerated spindle pole elongation. Our results showed no significant changes in spindle pole oscillation frequencies or amplitudes.
These findings suggest that SPD-1 does not play a major role in regulating spindle positioning forces in *C. elegans* zygotes, consistent with its specific localisation pattern.

Demonstrating that the depletion of three independent regulators, two of which are known to regulate microtubule rigidity, produces similar phenotypes is the most effective way to convince readers that altering microtubule rigidity causes spindle orientation defects independently of the effects on microtubule dynamics in the third regulator, ZYG-8.

While we agree that demonstrating similar phenotypes across multiple rigidity regulators would ideally strengthen our conclusions, our observations revealed important distinctions between these regulators. We found weak oscillation phenotypes in *ptl-1(RNAi)* embryos (likely due to low zygotic expression (Tintori *et al*., 2016)), and no oscillation changes in *spd-1(RNAi)* embryos.
Nevertheless, we believe these observations provide valuable information for the broader scientific community. We have therefore:

1. Added *spd-1(RNAi)* data to Supplementary Figure S14.
2. Included a new discussion paragraph (see below).

Figure S14 now displays the spindle-pole phenotypes upon *ptl-1(RNAi)* (panels A-B), previously shared, that we have complemented with spindle final position and orientation (panels C-D). *ptl-1(RNAi)* caused no significant perturbation in spindle final position/orientation. We have also added the phenotypes upon *spd-1(RNAi)* for spindle-pole oscillations (panels E-F), and spindle final position and orientation (panels G-H). *spd-1(RNAi)* produces no detectable changes in either spindle oscillations or final position/orientation.

The new paragraph in the Discussion (p. 17) reads as follows:

“To explore whether other microtubule rigidity regulators contribute to spindle positioning in *C. elegans* embryos, we examined two candidates: PTL-1, the sole nematode Tau homolog [117, 118], and SPD-1^{PRC1} [36]. PTL-1 depletion resulted in modest increases in oscillation amplitudes (34% anterior, 13% posterior, Figure S14A) and slight frequency decreases (9% anterior, 11% posterior, Figure S15B), alongside a 60% reduction in centring-to-damping corner frequency (Table 1). While these alterations resembled *zyg-8(RNAi)* phenotypes, their reduced magnitude likely reflects PTL-1's limited zygotic expression [119]. Similarly to *zyg-8(RNAi)* treatment, we observed no defects in spindle final position or orientation following

ptl-1(RNAi) (Figure S14C, D). Conversely, SPD-1 depletion produced no detectable changes in spindle oscillations (Figure S14E, F) or spindle final position and orientation (Figure S14G, H). This is consistent with SPD-1's spindle midzone localisation and absence from astral microtubules [59, 143, 144]. These comparative analyses highlight the unique role of ZYG-8 among microtubule rigidity regulators in controlling external forces on the spindle during *C. elegans* zygote division."

In summary, for my major concerns, the authors should look at potential changes in dynein activity and/or localization in ZYG8 perturbation to validate the "equal forces" assumption and complete the datasets on CLASP, MCAK, XMAP, and TAU with microtubule-associated phenotypes (cortical dynamics and shape analysis) to better discriminate which microtubule-level phenotypes are associated with spindle-level phenotypes. If the authors cannot provide all these new datasets, it should not prevent the publication of the manuscript, but the text would need to be amended to soften the conclusions regarding rigidity and discuss the limitations of the provided datasets.

We sincerely thank Reviewer #2 for this valuable summary of key concerns.

We have now provided additional data that do not indicate a major change in cortical pulling forces capable of explaining our observations.

We have expanded our analyses of CLASP and MCAK perturbations to include cortical microtubule dynamics (contact number/duration). These new data (Figures S6, S7) confirm that CLS-2^{CLASP} acts as a stabiliser (reduced contacts, shorter durations) and KLP-7^{MCAK} acts as a destabiliser (increased contacts, longer durations). Neither recapitulates *zyg-8* phenotypes. We have also complemented our analyses of spindle final position and orientation to include the phenotypes upon the perturbations of ZYG-9, SPD-2, CLS-2, and KLP-7. The comparisons using depletions of various MAPs have been better discussed (p. 15), to address a concern from the reviewer #1: "*While our comparisons using depletions of various MAP provide valuable insights into the role of microtubule dynamics in spindle oscillations and cortical microtubule contact phenotypes, we acknowledge some limitations. …*"

We have moderated our conclusions about PTL-1^{Tau}, not using it to stengthen ZYG-8's role in rigidifying microtubules. Instead, we have presented PTL-1 results in a Discussion paragraph alongside new results when we targeted SPD-1 (Figure S14).

Last, we have also discussed our conclusion about microtubule rigidity contribution, which also addresses Rewiever #3's concern (p. 16): "*While our data strongly implicate microtubule rigidity in spindle misorientation in zyg-8(or484ts) mutants, we cannot rule out the possibility of additional contributions from mild perturbations in microtubule dynamics.*"

<u>Minor comments</u>:

The manuscript is generally clearly written, and addressing the points below will further improve clarity and reproducibility

• The figure legends, and materials and methods sections do not specify the number of replicates.
In figure 1 for example "We tracked the centrosomes and analysed their positions in: (red) N

= 16 zyg-8(RNAi)-treated embryos and (black) N = 10 control RNAi embryos expressing GFP::TBB-2; (light green) N = 21 zyg-8(or484ts) mutants and (light blue) N = 17 untreated embryos, both at the restrictive temperature (Rest. T°) expressing GFP::TBB-2; (purple) N = 19 zyg-8 overexpressing embryos and (grey) N = 17 untreated embryos expressing mCherry::tubulin (Method M7)." Is the number of embryos specified here coming from a single experimental replicate or more? This problem is present throughout the manuscript in many, if not all, of the figures.
The authors should clarify the number of experimental replicates and indicate whether they consider it an appropriate number of animals analyzed per replicate, given the statistical analysis they are conducting.

We thank the reviewer for raising this point regarding experimental replication and statistical analysis.
For all quantitative analysis, our data were collected from at least two independent experimental sessions conducted under identical conditions. In further details, each experimental condition was examined across multiple biological replicates (independent experimental sessions) and each embryo was treated as an independent biological sample, consistent with established practices in *C. elegans* research. The total sample size (*N*) for each condition represents pooled data from these replicate sessions, and approximately equal numbers of embryos were analysed in each replicate. Indeed, the high reproducibility of our worm cultivation and treatment protocols, microscopy acquisition parameters and phenotypic measurements across replicates justifies pooling data from different sessions.
Statistical significance was assessed on embryo-level measurements, which we consider appropriate given the sample size per condition and the consistent phenotypic effect observed across experimental sessions.
To address this concern, we have clarified in the figure legends (when needed) that *N* values represent the total number of embryos analysed across all replicates and included a dedicated subsection in Methods (M15) p. 29 as follows.

"*M15- Experimental replication and Statistical analysis*
Each experimental condition was examined across multiple biological replicates (two to four independent experimental sessions) and each embryo was treated as an independent biological sample, consistent with established practices in *C. elegans* research. The total sample size for each condition, denoted *N*, represents pooled data from these replicate sessions. The high reproducibility of our worm cultivation and treatment protocols, microscopy acquisition parameters and phenotypic measurements across replicates justifies pooling data from different sessions.
Statistical significance was assessed on embryo-level measurements."

Similarly, for figures where authors measured subcellular objects that can be present in multiple copies per cell, such as microtubule comets, they should specify not only the number of objects but also the number of embryos from which the data are derived. For example, in the legend of figure 2 it says:" Growth rates of astral microtubules measured at (cross) anterior and (circle) posterior centrosomes: (B) (red) N = 27 comets from anterior centrosome and N = 23 comets from posterior centrosome in 10 zyg-8(RNAi)-treated embryos, and (black) N = 26 comets from anterior centrosome and N = 24 comets from posterior centrosome in 8 control RNAi embryos;" I can imagine that one single spindle could contain hundreds or thousands of growth events from which a subset will be satisfactory for analysis. So, the numbers of comets here seem small to me. Is this number of comets coming from one single embryo or from the

aggregate of multiple experiments? How many comets are analyzed per spindle, then, and is it appropriate?

We thank the reviewer for raising this point regarding the quantification of subcellular objects and understand the need for clarity in our analytical approach.
Consistent with our previous response regarding replication, all quantitative analyses of microtubule comets were conducted across at least two to three independent experimental sessions for each condition.
To clarify our methodology, in particular the sampling strategy, approximately 5 comets per embryo were analysed. With about 10 embryos collected per condition, this resulted in about 50 comets analysed in total per condition. For comparisons between anterior and posterior centrosomes, we analysed ~25 comets per centrosome (2-3 comets per centrosome per embryo).
We understand the reviewer's concern regarding the relatively small number of comets analysed compared to the thousands of growth events occurring in each embryo. This apparent discrepancy arises from our stringent selection criteria, which are essential for accurate measurements. Firstly, we only analysed comets that remained on the focal plane for ≥4 seconds. This criterion is critical to avoid errors in growth speed estimation that would arise from analysing comets moving in three dimensions. Since our imaging was conducted on a single focal plane (now clarified in the revised manuscript), this requirement substantially reduced the number of analysable comets. Secondly, to further minimize measurement errors, we exclusively selected comets whose growth occurred when centrosomes were stationary. This restriction explains our focus on metaphase (when centrosome movement is minimal) and further reduced the pool of analysable comets. To be noticed that this analysis was derived from the one presented by Srayko *et al.*, who quantified a similar number of comets per embryo due to the same selection criteria.
To address these concerns, we have updated all relevant figure legends and have expanded Method section M8 p. 23 to include detailed sampling methodology and explanation of our selection criteria to ensure measurement accuracy, as follows (addition shown in bold):

"*M8 - Characterisation of astral microtubule growing and nucleation rates*
…/… We measured microtubule dynamics during metaphase **using single-focal-plane imaging**.
To measure the growth rates, we first denoised the movies using Kalman filtering, with the gain set to 0.5 and the initial noise estimate set to 0.05 [188] (Figure 2A). Next, we traced a line along the comet trajectory in the pre-processed movie, restricting to comets that remained visible **in the focal (XY) plane** for at least 10 frames (4 s) **to exclude comets growing in the three dimensions (XYZ)**. We generated a kymograph from the traced line to assess comet displacement velocity. **We analysed ~50 comets per condition (distributed across ~10 embryos, with ~5 comets analysed per embryo), ensuring balanced sampling from both anterior and posterior centrosomes (~25 comets per centrosome) when possible.**"

This issue is present in multiple figures, notably Figures 2, 4, and 6. For these figures (and all others where it is appropriate), the authors should indicate the number of animals used per replicate, the number of objects analyzed per replicate, and the number of replicates.

We have revised all relevant figure legends to explicitly report the number of embryos analysed across all replicates for each convidtion, or/and the number of objects (e.g., comets) per condition. Additionally, we have expanded Method sections M15 and M8 to clarify sampling

strategy and experimental replicate number. These revisions ensure full transparency regarding our experimental design and statistical approach while maintaining clarity in the figure presentations.

• Continuing with the topic of my previous comment, I think authors should strongly consider representing the data as SuperPlots (https://doi.org/10.1083/jcb.202001064). This will greatly help the reader evaluate the spread in the data, the number of replicates used, and the number of individuals per replicate, allowing them to easily assess the appropriateness of the experimental design. I am thinking about Figure 1 C and D, Figure 2 B, C, and D, Figure 3 B, C, D, G, and H, Figure 4 F and H, Figure 6 E, F, G, and H, and Figure 8 C and D.

We appreciate the reviewer's suggestion to use SuperPlots for data visualisation, as this approach can indeed provide valuable insights into data distribution and experimental design. We have carefully considered this recommendation in light of our specific experimental context and established practices in *C. elegans* research. For the current study, we have chosen to use beeswarm plots combined with box plots for the following reasons:

1- These visualizations effectively show both the spread of our data and key statistical measures.
2- In our *C. elegans* embryo system, we typically observe low biological variability between replicates. This low variability means that the additional information provided by SuperPlots would not substantially alter the interpretation of our results.

• For the microtubule shape analysis, it would be a great help to the reader if the authors could provide a side-by-side of the spindle image and the resulting traces. The authors provide only a single side-by-side example in Figure S8, without indicating whether this represents a CTRL or perturbed embryo. I would suggest providing a few side-by-sides for each condition analyzed. This will showcase the methodology and allow readers to perform a visual validation.

We thank Reviewer #2 for this valuable suggestion to enhance the visualisation of our microtubule shape analysis methodology.
As noted in the legend, the original Figure S8 serves primarily as a methodological demonstration of our analysis pipeline, using an untreated (control) embryo as an example. This figure was designed to illustrate our general approach rather than compare experimental conditions.
To directly address the reviewer's request, we have created a new supplemental figure (Figure S11) that provides side-by-side comparisons of microtubule shape images and their corresponding curvature traces for each experimental condition. We have used the same embryos previously displayed in Figure S7D,E to maintain consistency. We have added some quantitative context by indicating the microtubule numbers analysed and the total pixel numbers processed for each exemplar shown.
Additionally, we have merged relevant panels from the original Figures S7 and S8 to create a new supplemental figure (Figure S8) focused specifically on microtubule shape quantification methods. We have also explicitly labeled panels E-J in this new figure to indicate they derive from an untreated embryo.

• "A previous study showed that microtubules in axonal growth cones were more often strongly

curved upon depletion of the Doublecortin-family proteins DCX and DCLK1." This statement should be supported by a reference.

We thank the reviewer for identifying this oversight. We have now added the appropriate reference to the main text to support our statement:

Jean, D.C., P.W. Baas, and M.M. Black, *A novel role for doublecortin and doublecortin-like kinase in regulating growth cone microtubules.* Human molecular genetics, 2012. **21**(26): p. 5511-5527.

• "Figure 7: Depleting ZYG-8 weakens highly the cortical pushing events." This figure title needs correcting for its English.

We thank Reviewer #2 for identifying this linguistic issue. We have revised the title of Figure 7 to improve clarity and grammatical correctness. The corrected title now reads:

"Depletion of ZYG-8 significantly weakens cortical pushing events"

• Considering SPD-1/Ase1/PRC1/MAP65-1, the text states "Research, particularly in neuronal systems, has identified two major protein families involved in regulating microtubule rigidity […] the Doublecortin family, […] and the MAP2/MAP4/Tau superfamily." This statement overlooks the fact that the SPD-1/Ase1/PRC1/MAP65-1 family is involved in microtubule rigidity, albeit not in neuronal systems, and has been proposed to affect microtubule rigidity independently of microtubule dynamics. See https://doi.org/10.1091/mbc.e13-03-0141 or https://doi.org/10.7554/eLife.53807, for example. The statement, or the paragraph, could mention this family of proteins.

We thank the reviewer for this important observation regarding the SPD-1/Ase1/PRC1/MAP65-1 family. We fully acknowledge that our original text overlooked this critical family of microtubule regulators, and we have now revised the introduction to include this information.
The two updated paragraphs now reads (additions/modifications shown in bold):

p. 3: "Research **across various biological systems** has identified **three** major protein families involved in regulating microtubule rigidity: the Doublecortin family (including DCX and DCLK1), the MAP2/MAP4/Tau superfamily, **and the SPD-1/Ase1/PRC1/MAP65 family** [16, 18, 34-36]. **While the latter family plays crucial roles in non-neuronal contexts, the first two have been primarily studied in neuronal systems.** …/… **In contrast to Doublecortin and Tau proteins, MAP65/Ase1, by binding to individual microtubules and microtubule bundles, decreases their flexural rigidity [36]. Upon crossing-linking antiparallel microtubules, proteins of this family favour the formation and stabilisation of microtubule bundles [36, 45-47]. Importantly, unlike the SPD-1/Ase1/PRC1/MAP65 family, both the Doublecortin and Tau families can** also regulate microtubule dynamics, making it challenging to disentangle their roles in mechanical versus dynamic regulation. …/…"

p. 4: "Previous studies on cell division involving the targeting of proteins from the Doublecortin, Tau, **and PRC1** families revealed several defects. These included mitotic

spindle mispositioning, abnormalities in spindle formation and integrity, and impaired cell cycle progression **[51-60]**. …/… **Proteins from the PRC1 family were also suggested to regulate mitotic spindle mechanics [45, 47].** Thus, targeting proteins **from these three families** can lead to a wide range of meiotic and mitotic disturbances, although the underlying mechanisms remain unclear. …/…"

The following references were added to support our statement:

[36] Portran D, Zoccoler M, Gaillard J, Stoppin-Mellet V, Neumann E, Arnal I, et al. MAP65/Ase1 promote microtubule flexibility. Mol Biol Cell. 2013;24: 1964–1973. doi:10.1091/mbc.e13-03-0141

[45] Tai E, Henglein A, Alfieri A, Saxena G, Forth S. Insights into the role of phosphorylation on microtubule cross-linking by PRC1. Mol Biol Cell. 2025;36: ar34. doi:10.1091/mbc.E24-12-0565

[46] Braun M, Lansky Z, Fink G, Ruhnow F, Diez S, Janson ME. Adaptive braking by Ase1 prevents overlapping microtubules from sliding completely apart. Nat Cell Biol. 2011;13: 1259–1264. doi:10.1038/ncb2323

[47] Suresh P, Long AF, Dumont S. Microneedle manipulation of the mammalian spindle reveals specialized, short-lived reinforcement near chromosomes. Akhmanova A, Surrey T, Redemann S, Shimamoto Y, editors. eLife. 2020;9: e53807. doi:10.7554/eLife.53807

[59] Verbrugghe KJ, White JG. SPD-1 is required for the formation of the spindle midzone but is not essential for the completion of cytokinesis in C. elegans embryos. Current Biology. 2004;14(19):1755-60. doi:

[60] Mollinari C, Kleman J-P, Jiang W, Schoehn G, Hunter T, Margolis RL. PRC1 is a microtubule binding and bundling protein essential to maintain the mitotic spindle midzone. Journal of Cell Biology. 2002;157(7):1175-86. doi: 10.1083/jcb.200111052.

Reviewer #3:

Cueff et al., 2025 report a microtubule stiffening role for ZYG-8 in regulating mitotic spindle orientation. Using 3 complementary methods, the authors disrupt the fuction of zyg-8/DCLK1, the C. elegans Doublecortin and find incorrect mitotic spindle positioning, as reported earlier. In the zygote, ZYG-8 modestly promotes microtubule growth and limits nucleation. Further analysis showed changes in spindle pole oscillations and microtubule cortical-contact behaviour in Zyg-8 depleted cells. ZYG-8 depletion or mutation led to curved microtubules and increased cortical life-time. Using simulations, the authors show that that reduced rigidity can increase cortical lifetimes. They correlate microtubule softening in zyg-8 depleted embryos and mutants with decreased spindle centering, which may be linked to increased spindle oscillations. During anaphase the authors find spindle poles travelling closer to cell periphery associated with mispositioned spindles. Overall, most observations have high quality images, although the display of measurements (graphs) can be strengthened. While its possible that microtubule curvature may play a significant role in regulating Zyg8 disruption induced spindle positioning phenotype, the evidence for ruling out microtubule dynamics is somewhat weak. This is at least in part because it's difficult to rigorously correlate spindle movements with 3D growth rates of microtubules - at multiple regions of the spindle - through time. The authors could manage this knowledge gap with a balanced discussion rather than omitting the role of microtubule dynamics altogether.

In general, I find this paper very strong with high-resolution movies; some graphs and discussion can be strengthened.

We sincerely thank the reviewer for the thorough and constructive evaluation of our manuscript. We appreciate both the positive assessment of our work and the insightful suggestions for improvement, which have helped us strengthen our presentation and discussion.
In response to the reviewer's valuable comment regarding microtubule dynamics, we have revised our discussion to clarify that we do not exclude a role for ZYG-8 in regulating microtubule dynamics. Our data suggest that ZYG-8's primary contribution to spindle positioning and orientation—particularly under conditions of elevated cortical pulling forces—appears to be mediated mostly through its effects on microtubule mechanics rather than dynamics. This revised discussion (see below) now provides a more balanced perspective that acknowledges the complexity of ZYG-8's functions while maintaining our core conclusion about the dominance of mechanical regulation in our experimental context.

Major points:

1) Cueff et al., indicate "DCX proteins bind to microtubules and are thought to stabilise or rigidify them." Doublecortin in human cells induces mitotic microtubule catastrophe – they show its relevance to glioblastoma (Santra J Neruochem 2009). It will help readers if the authors discuss the past DCX studies in the context of new findings. For example, what is the relationship between microtubule stiffening and microtubule dynamics (or catastrophe). This is particularly important as the authors propose implications for cancer therapy, by stating doublecortin is deregulated in human cancer.

We thank the reviewer for raising this point regarding the relationship between DCX function, microtubule dynamics and rigidity.
We have incorporated the suggested reference (Santra *et al.*, 2009) into our introduction, specifically in the paragraph dedicated to cell division studies of the DCX family.

The majority of previous studies on DCX family proteins have focused primarily on their roles in spindle assembly and morphology—rather than spindle positioning and orientation (the central focus of our current work)—with the notable exception of Gönczy *et al.* (2001). In these studies, Doublecortin's spindle-associated functions have frequently been linked to molecular motor activity, potentially through indirect mechanisms. However, to our knowledge, no studies to date have directly examined the relationship between molecular motor binding/activity and microtubule stiffness, which limits our ability to fully develop this discussion—though we fully acknowledge its scientific interest.
While several publications report alterations in spindle microtubule morphology following Doublecortin protein depletion or overexpression, these phenotypes can typically be attributed to spindle assembly defects, making it challenging to specifically attribute them to changes in microtubule rigidity. In contrast, Czajkowski *et al.* (2024) clearly demonstrate modifications of spindle microtubule shape upon ZYG-8 targeting in assembled bipolar meiotic spindles, a phenotype we discuss in relation to our new findings in the penultimate paragraph of the Discussion.

We agree with the reviewer that exploring the relationship between microtubule mechanics and dynamics represents an important scientific question. However, the current lack of studies directly addressing this relationship—particularly in the context of catastrophe events—makes it difficult to substantively develop this discussion within the scope of our present study.

2. Abstract rules out microtubule growth defects as a reason for change in oscillations. But Discussion section indicates "… a dual role for ZYG-8DCLK1 during mitosis, involving the control of both microtubule rigidity and dynamics." Ruling out of microtubule dynamics in spindle movements appears to be based on indirect outcomes. For example, through comparing ZYG-9 partial depletion that achieves a partial reduction in microtubule growth rate: "16% reduction in microtubule growth rate, comparable to that observed in zyg-8 mutants (Figure S4B).". This type of indirect comparison does not rule out the possibility of 15% change in microtubule growth in zyg-8 cells have a role in addition to changes in microtubule rigidity. This is important because disruption of PTL-1/Tau known to reduce microtubule rigidity causes milder centring defects compared to zyg-9 loss.

We thank the reviewer for raising this point regarding the potential contribution of microtubule dynamics to the observed phenotypes. We fully acknowledge that our initial phrasing may have created ambiguity, and we have taken steps to clarify this throughout the manuscript.

Our intention has not been to rule out a contribution of microtubule dynamics *per se*, but rather to demonstrate that alterations in microtubule dynamics <u>alone</u> are not sufficient to account for the specific perturbations we observed upon targeting *zyg-8*.

We have now strengthened our quantitative comparisons by including additional data on spindle final position and orientation across multiple conditions that specifically impair microtubule dynamics: *zyg-9(RNAi)* (Figure S4E, F), *spd-2(RNAi)* (Figure S5E, F), *cls-2(RNAi)* (Figure S6D, E), and *klp-7(RNAi)* (Figure S7D, E).
These analyses reveal distinct phenotypes compared to *zyg-8* mutants, supporting our conclusion that *zyg-8* mutation produces effects beyond those attributable to dynamics alone (now addressed in Section 5 of the Results, p. 14). This have confirmed an additional contribution from ZYG-8 to explain these phenotypes.

We have thus proposed that microtubule rigidity plays a critical role in this process, acting cooperatively with microtubule dynamics. To this end, we wrote in our abstract "*<u>These changes could not be fully explained</u> by previously reported alterations in microtubule dynamics*".
Throughout the manuscript, we have carefully checked where some ambiguity could exist and revised the text where needed as follows.

<u>Abstract</u> (p. 2):
"Importantly, **these phenotypes** could not be fully explained by previously reported alterations in microtubule dynamics, **suggesting an additional mechanistic origin**."

<u>Section 1</u> (p. 7):
"Together, these findings suggested that ZYG-8's role in limiting microtubule nucleation alone ~~did not~~ **was not sufficient to** account for the anaphase spindle oscillation phenotypes observed upon targeting *zyg-8*."

<u>Section 2</u> (p. 9):

"Overall, our data indicated that the *zyg-8*-associated perturbations in spindle-pole oscillations and cortical microtubule dynamics could not be **fully attributed to** ~~explained solely by~~ alterations in microtubule dynamics **alone**. …/… We asked whether the observed perturbations in spindle-pole oscillations and cortical microtubule behaviour could **additionally** result from altered microtubule rigidity upon targeting *zyg-8*."

Discussion (p. 14):
"**However**, the modest changes observed in astral microtubule dynamics in the cytoplasm upon *zyg-8* targeting, as well as its potential role in stabilising microtubules, are **not** ~~un~~sufficient to **fully** explain the phenotypes related to spindle pole oscillations during anaphase, **spindle final position and orientation**, or the altered behaviour of astral microtubule contacts at the cell cortex"

Instead of the sentence "*We propose that ZYG-8 also regulates microtubule rigidity, and that this function is the strongest contribution to the phenotype*." that was overly concise and lacked sufficient context, we have expanded our discussion by adding two new sections at distinct locations:

p. 15: "**Second, cumulative effects of multiple perturbations may contribute to the phenotypes observed upon *zyg-8* targeting. Nonetheless, taken collectively, our set of experiments suggests that alterations in microtubule dynamics alone cannot fully recapitulate the spindle-pole oscillation variations, spindle final position defects, and cortical microtubule contact phenotypes observed upon *zyg-8* targeting. This supports our conclusion that ZYG-8 plays an additional role beyond simply regulating microtubule dynamics."**

p. 16: "**Our data suggest that, while ZYG-8 contributes to microtubule dynamics, its primary role in regulating spindle positioning and orientation is mediated through modulation of microtubule rigidity, particularly under conditions of elevated cortical pulling forces (Figure 8A-D). This mechanical function likely represents the dominant mechanism accounting for the observed phenotypic effects, with alterations in microtubule dynamics playing a secondary role**."

Additionally, we have expanded our discussion p. 16 to provide a more nuanced perspective:
"While our data strongly implicate microtubule rigidity in spindle misorientation in *zyg-8(or484ts)* mutants, we cannot rule out the possibility of additional contributions from mild perturbations in microtubule dynamics."

3. Impaired pushing forces have been reported following Mark2/Par1b loss (see below): Excessive defects with spindle-centring and spindle oscillations have been observed in human cells lacking MARK2/Par1 kinase (Zulkipli et al., 2018 Journal of Cell Biology) or following MARK2 inhibitor treatment (Dang et al., 2023 Journal of Cell Biology). They have been reported to be independent of cortical dynein. These published works should be either introduced in introduction, or at least considered in discussion.

We acknowledge that it is of interest to include this litterature in our article and thank the reviewer for this suggestion. We have added these works in our discussion in the paragraph dedicated to the cortical pushing force relevance beyond the nematode's first division.

We have supplemented the discussion p. 18 as follows (addition shown in bold):

"We foresee that the essential role for pushing extends beyond the nematode's first division and may be relevant to other asymmetric divisions and maybe further. …/… **Furthermore, spindle orientation and centring can occur through cortical-dynein-independent mechanisms, such as those involving MARK2/Par1 kinase [29, 161].** Consequently, the traditional canonical model alone cannot account for spindle orientation in various contexts. **Consistent with these observations,** we propose that the cortical pushing force contributes to this phenomenon and that the flexural rigidity of microtubules must be sufficiently high. …/…"

4. Figure 5 images show a clear difference in microtubule structure, but Figure 6 curvature measurements need statistical significance. Curvature measurement method can help reader. I would suggest merging these two figures so that it easier to see the graphs on the side of the images.

We thank the reviewer for these valuable suggestions to improve the presentation of our microtubule curvature data.
We understand the reviewer's suggestion to merge Figures 5 and 6. However, these figures present distinct datasets obtained through different experimental approaches:

- Figure 5 shows live imaging of embryos.
- Figure 6 presents quantitative curvature measurements from fixed embryos (with representative images provided in Figure S11).

To improve clarity, we have added the following statement to the Figure 6 legend: *"Characterization of microtubule shapes in fixed embryos analysed using the image-processing pipeline depicted in Figure S8."*
We believe that revisions addressing Reviewer #2's request—to include curvature maps alongside Figure S11 images—have also helped clarify the curvature measurements (see answer p. 21).

Regarding the reviewer's observation about high local curvatures in Figure 5 about live mutants, we confirm this aligns with panels C & D of Figure 6 (fixed-mutant data showing high curvature). Besides, our new Figure S9, which now includes live images of *zyg-8(RNAi)* embryos demonstrating higher curvatures in *zyg-8(RNAi)* vs. control RNAi is consistent with Figure 6A,B. Figure S9 also showed reduced curvatures in *zyg-8(RNAi)* live embryos compared to *zyg-8* live mutants from Figure 5, which is consistent with quantitative results in Figure 6.

6. Figure 6: sample sizes are somewhat low. Particularly 6A has only 5 samples.

We understand the concern about the apparently low sample numbers, and we would like to clarify that these numbers represent z-projected stacks per condition, not individual measurements.
Each analysed stack contains 15-70 individual microtubules, and curvature measurements are taken at every pixel along each microtubule. This results in a substantially larger dataset than the stack numbers might suggest.
Given the technically demanding nature of these experiments (requiring precise embryo fixation, proper embryo shape, high-resolution imaging, and meticulous image processing), we prioritised data quality and consistency over increasing the number of biological replicates.

The current dataset provides statistically robust curvature measurements while maintaining rigorous experimental standards.
To better communicate the actual scope of our analysis, we have made the following additions:

- In the Methodological Figure S8, panel H now indicates the number of microtubules analysed per condition and the panel J specifies the total number of pixels from which curvature measurements were derived.
- In Figure S11, we have included the same quantitative information (microtubule numbers and pixel counts) for all four displayed conditions.

These additions provide readers with a more accurate representation of our dataset's true size and statistical power.

5. In Figure 7a – the model, the three sections of the cartoon is somewhat unclear: in section 3,why would the rigid microtubule that started depolymerising in section 2 continue to be in depolymerising state (This microtubule marked in green could be reversing to growth phase or remaining as such as there is no cortical contact push after the initiation of depolymerisation in stage 2).

We thank Reviewer #3 for raising this point regarding the clarity of our model in Figure 7A. We recognize that two key aspects of astral microtubule dynamics in the *C. elegans* zygote were not sufficiently explained.

The reviewer correctly identifies that our model assumes continued depolymerization of the rigid microtubule in section 3. This assumption is based on well-established observations that rescue events of depolymerising astral microtubules are rare in the *C. elegans* zygote cytoplasm. To clarify this, we have added the following statement to the Figure 7A legend: *"Note that rescue of depolymerising astral microtubules is rare in C. elegans zygote cytoplasm, supporting continued depolymerisation."*

We acknowledge that the original schematic may have misrepresented the temporal scale of depolymerisation events. Specifically, the time window between the catastrophe events of the softened microtubule (#a) and the rigid microtubule (#b) is very short (a few hundred milliseconds). This brief duration minimizes the probability of rescue events. To address this, we have modified the schematic to adjust the relative lengths of microtubules in stage 3 to better reflect the short time range between events.

6. The authors propose in abstract "..sufficient microtubule rigidity is essential for generating effective cortical pushing forces, which contribute to centring mechanisms..."

I agree with the author's statement in discussion that it is "important to explore the mechanisms by which ZYG-8 rigidifies the microtubules." However, the sentence in abstract quoted above is built on several correlative evidence, or lack of severe phenotypes, by studying different types of depletions and mutant conditions.
I would recommend a cautious model that does not exclude any of the moderate phenotypes, particularly those that are not directly measured (for example, microtubule catastrophe rates) or not highly striking (for example, changes to microtubule growth).

We fully acknowledge that our study incorporates correlative evidence, and we have taken care to present our conclusions with appropriate nuance.

Our statement that *"sufficient microtubule rigidity is essential for generating effective cortical pushing forces, which contribute to centring mechanisms"* is based on multiple lines of converging evidence:

- Graduated Phenotypes:

Microtubule rigidity is more substantially reduced in *zyg-8* mutants compared to *zyg-8(RNAi)* embryos. This is consistent with a greater decrease in centring forces (as measured by positional fluctuation assays). These findings align with the gradual phenotypes observed in spindle pole oscillations.

- Force Balance:

Depletion of GPR-1/2 in *zyg-8* mutants restores proper spindle positioning, suggesting an imbalance in pulling/pushing forces in the mutant. This directly supports our mechanistic model.

Regarding the perturbations in microtubule growth alone (15% reduction in growth rate) that cannot fully explain the phenotypes observed in *zyg-8* mutants, we have strengthened our data on final spindle position and orientation following *zyg-9(RNAi)* (Figure S4E, F). We have also revised the discussion to explicitly include potential synergistic effects between microtubule dynamics and rigidity (see comment above).

While we did not directly measure catastrophe rates, we have strengthened our correlative evidence as suggested by Reviewer #2 by adding complementary experiments, namely microtubule contact counts and durations at the cell cortex following *cls-2(RNAi)* and *klp-7(RNAi)* (Figure S6F, G and Figure S7F, G). These data do not support a stabilisation role for ZYG-8 in the zygote. Additionally, we have analysed final spindle position and orientation for these conditions, which remain unaffected (Figure S6D, E and Figure S7D, E).

In section 5 of the Results (p. 14), we have supplemented the text with the following paragraph to introduce these additional experimental data about spindle final position and orientation upon various MAP depletions:

"Importantly, we quantified spindle final position and orientation across all experimental conditions in our study. The selective perturbation of microtubule growth via *zyg-9(RNAi)*—which induces changes in growth rates comparable to those observed in *zyg-8(or484ts)* mutants—did not affect spindle final position or orientation (Figure S4E, F). Moreover, specific impairments in microtubule nucleation (SPD-2 depletion; Figure S5E, F), microtubule stability (CLS-2 depletion; Figure S6D, E), or microtubule depolymerisation (KLP-7 depletion; Figure S7D, E) similarly failed to perturb spindle final position or orientation. Together, these observations further supported our conclusion that reduced microtubule rigidity, rather than altered microtubule dynamics, is the determining factor of spindle misorientation in *zyg-8(or484ts)* mutants."

To directly address the reviewer's concern, we have revised the abstract to adopt more nuanced language:

"We propose that sufficient microtubule rigidity is essential for generating effective cortical pushing forces**, potentially in synergy with other microtubule properties,** which contribute to centring mechanisms that ensure accurate spindle orientation in late mitosis."

Minor points

1. Please explain biophysical assays in results sections. Reference to such assays are found in abstract and introduction but not within results.

We sincerely thank the reviewer for this suggestion to clarify the presentation of our biophysical assays in the Results section. We recognize that while these assays were indicated in the Abstract and Introduction, their specific designation as biophysical methods was not explicitly stated in the Results section.
We have supplemented the main text with clearer designations and expanded explanations of the biophysical assays (new additions/modifications shown in bold):

p. 11: "Next, we applied the DiLiPop analysis, **a biophysical method that enables** to assess cortical pulling and pushing events during anaphase. **Our previous work has shown** that two **distinct** populations of astral microtubules reside at the cell cortex, **disentangled by** statistical analysis of cortical contact durations [80] (Method M10). By genetically perturbing either pulling or pushing cortical forces, we **previously** associated the short-lived population with pulling events and the long-lived population with pushing [80]."

p. 12: "To do this, we used a **biophysical** assay previously developed **by our laboratory** focussing on the spindle positional micro-movements acquired at high frame rate. We extracted the mechanical fingerprint of these micro-movements along the transverse axis during anaphase. By fitting a second-order model to the experimental spectrum, **we derived three mechanical parameters that characterise spindle position mechanics: the diffusion coefficient D and two corner frequencies f_c and f_0** [25] (Method M14)."

2. Unclear how microtubule growth rates were observed (was it between 10 consecutive time points for comets moving in XY or XYZ .

3. Please explain why it needs to be measured 50 times: "We repeated the measurement approximately 50 times for each condiHon across about ten embryos (about 25 comets per centrosome)."

We thank the reviewer for raising these two methodological questions (2, 3), which provide an opportunity to clarify our approach to measuring microtubule growth rates.

To address the reviewer's first concern about how microtubule growth rates were observed, we performed imaging on a single focal plane (XY), which necessitated careful selection of microtubules for analysis. To minimize errors in growth speed estimation that could arise from three-dimensional movement, we restricted our analysis to EB1 comets that remained continuously visible in the focal plane and were tracked for at least 10 consecutive frames (4 seconds). We have now added these clarifications to the revised manuscript to improve the transparency of our methodology (see previous answer to comment from Reviewer #2 p. 20).

Regarding the reviewer's question about why measurements were repeated 50 times per condition, our experimental design was structured to ensure robust statistical power and biological relevance. Around 5 comets were analysed per embryo to account for variability

within individual embryos. Data were collected from 10 embryos per condition, distributed across at least 2 independent experimental sessions. This resulted in 50 measurements per condition. This also provides consistency with prior work, since similar measurement numbers were used in (Srayko *et al*., 2005).

4. Figure 3E needs scale bars.

We thank the reviewer for pointing out this important detail. We have now addressed this by adding scale bars to Figure 3E to provide proper spatial reference for the images presented. We have also included scale bars in Figure S5A as well, as this figure contains similar image panels.

5. Are the spindle mispositioning phenotypes most prominent in anaphase? Would a microtubule curvature induced phenotype reduce with reducing microtubule length?

We thank Reviewer #3 for raising these insightful questions regarding the timing of spindle mispositioning phenotypes and their potential dependence on microtubule length.

Our observations indicate that spindle mispositioning phenotypes are specifically associated with anaphase in *zyg-8* mutants. This is demonstrated in Figure 8, where time-lapse imaging shows that at anaphase onset (time point 0:00), spindles are correctly positioned at the cell center in both untreated controls and *zyg-8* mutants at restrictive temperature.
To further emphasize this temporal specificity, we have added additional data to Figure 8 (panels C and D), showing spindle position and orientation at mid-metaphase and the metaphase-to-anaphase transition.
The anaphase-specific nature of these phenotypes can be explained by differences in force dynamics between metaphase and anaphase. During metaphase, cortical pulling forces are relatively low (Pécéraux et al., 2006; Garzon-Coral et al., 2016; Bouvrais et al., 2021), so reduced microtubule rigidity (and consequently reduced cortical pushing forces) does not significantly disrupt the pulling/pushing force balance. During anaphase, cortical pulling forces increase substantially in the *C. elegans* zygote, making sufficient cortical pushing force critical to counterbalance the enhanced pulling. We validated this mechanism by demonstrating that reducing cortical pulling forces rescues the mispositioning/misorientation phenotypes during anaphase.

We have clarified the timing of spindle misorientation in the text at the end of the introduction p. 5 as follows:

"Our results demonstrated that ZYG-8 is needed to maintain the correct spindle orientation during ~~mitosis~~**anaphase**, primarily by permitting high centring forces. Importantly, these centring forces depend on microtubule flexural rigidity, **and their elevated levels specifically counterbalance the increased cortical pulling forces observed during anaphase**."

We have also complemented the Results section (p. 13) to better highlight the specific period of the position and orientation defects as follows:

"**Notably, spindle mispositioning and misorientation in *zyg-8(or484ts)* mutants were restricted to anaphase, as we observed no significant differences in spindle position or orientation between mutants and untreated embryos at either mid-metaphase or**

anaphase onset (Figure 8C, D). These observations suggested that the anaphase-specific increase in cortical pulling forces may contribute to the observed defects."

Regarding the reviewer's question about potential length dependence of curvature phenotypes, cortical pushing force is indeed inversely proportional to microtubule length. We could expect that microtubule curvatures would be less pronounced in longer microtubules.

6. Would microtubules experience reduced tortuosity as they are being pulled compared to those that are not being pulled by cortical dynein?

We sincerely thank Reviewer #3 for raising this interesting question regarding the relationship between microtubule tortuosity and cortical pulling forces.
Microtubules undergoing active pulling by cortical dynein would theoretically experience tensile forces, which should minimize bending and thus reduce tortuosity. Conversely, microtubules subject to pushing forces would experience compressive forces, making them more prone to buckling and increased tortuosity.
Our time-lapse imaging (e.g., Movies S5-S7) and fixed-cell analysis (Figure S11) reveal that some microtubules contacting the cell cortex appear notably straight, which we interpret as potential engagement in pulling events. Other microtubules display visible curvature, consistent with compressive forces during pushing events.

7. The observations does not rule out asymmetric regulation of cortex reaching microtubules from one pole but not the other "Thirdly, we show that in zyg-8(or484ts) mutants, astral microtubules are still able to contact the opposite cortex (Figure 5A, Movies S5 and S6). This rules out a model in which spindle misorientation arises from a failure of microtubules to reach the cortex…,".

We appreciate Reviewer #3's important observation regarding the potential for asymmetric cortical microtubule interactions. This comment provides an opportunity to clarify our interpretation of the data and the mechanistic implications.
Previous studies have indeed proposed that spindle misorientation can result from failed cortical contact by astral microtubules, as cortical forces exerted by these microtubules are critical for proper spindle positioning and orientation (Saleh *et al*., 2023). Our own prior work has similarly emphasized that cortical access of astral microtubules plays a key role in spindle positioning regulation (Bouvrais *et al*., 2018).
In *zyg-8* mutants during anaphase, one might hypothesize that spindle misorientation could arise from asymmetric microtubule-cortex interactions, particularly given that the spindle is positioned closer to one cortex. This proximity could potentially result in increased centrosome-cortex distance on the opposite side, and microtubules being too short to establish cortical contacts. However, our live imaging of *zyg-8* mutants (Figure 5A, Movies S5 and S6) clearly demonstrates that astral microtubules maintain the ability to contact both cortical sides. Therefore, we can exclude the possibility that spindle misorientation in these mutants results from a failure of microtubules to reach the cortex.
Importantly, the *C. elegans* zygote exhibits axis-specific cortical organisation with the anterior-posterior axis showing asymmetry (e.g., PAR protein localisation). However, the transverse axis (up-down) does not display such cortical asymmetry. Given this biological context, it is highly unlikely that *zyg-8* mutants experience asymmetric regulation of microtubule-cortex interactions along the transverse axis.

8. Figure 8 panel B show prominent astral microtubules on the anterior spindle – please comment.

We thank the reviewer for bringing this observation to our attention. After careful consideration of potential explanations—including imaging artifacts, chromosome-induced localisation effects, or other biological factors—we examined additional *zyg-8* mutants at the restrictive temperature. We did not observe this pattern consistently across replicates (only 3 out of 21 embryos).
To ensure clarity and prevent potential misinterpretation, we have replaced this image with an alternative embryo that more accurately reflects the typical phenotype observed in our experiments.

9. Include Zulkipli et al., 2018 while discussing metaphase only phenotypes here: "To date, cortical pushing forces have been proposed to contribute to spindle centring during metaphase [25, 27]."

We thank Reviewer #3 for this valuable suggestion to include Zulkipli *et al*., 2018 in our discussion of metaphase-specific phenotypes. We fully agree that this reference strengthens our argument regarding cortical pushing forces, and we have incorporated it into our manuscript in the following ways:

- We have now included Zulkipli *et al*., 2018 in our discussion of metaphase phenotypes, modifying the relevant passage to read:
  p. 17: *"To date, cortical pushing forces have been proposed to contribute to spindle centring during metaphase [25, 27, 29]."*
- We have also incorporated this reference into our introduction to bolster the statement about pushing forces:
  p. 3: *"...for generating pushing forces against the cell periphery that contribute to spindle or nucleus positioning [23-30] ..."*

To further strengthen this section, we have added another key reference:
Zhao T, Graham OS, Raposo A, St Johnston D. (2012) Growing microtubules push the oocyte nucleus to polarise the Drosophila dorsal-ventral axis. *Science* 336: 999-1003. doi:10.1126/science.1219147

10. These discussion sentences in quotes below are somewhat unclear and feel stretched considering the protein is involved in embryonic development and cell division – the discussion is strong without these extended therapeutic plans. "Given that DCLK1 is frequently deregulated in various solid tumours (e.g., those from the colon, pancreas, kidney and breast), this suggests its involvement in a common process such as cell division [154-157]. Additionally, as the proteins of the Tau family are also found to be deregulated or mutated in cancers, targeting microtubule rigidity may represent a promising approach for future cancer treatments [158-160]. This strategy could potentialise existing therapies targeting microtubule dynamics, which ooen suffer from side effects and are limited by cancer cell resistance."

We appreciate Reviewer's comment regarding the discussion of therapeutic implications in our manuscript. We understand the concern that these sentences might extend beyond the immediate scope of our findings on embryonic development and cell division. Our intention

was to provide a brief perspective on potential broader implications, rather than to propose concrete therapeutic strategies.
To address this concern, we have revised this section, notably to maintain a more measured tone regarding potential therapeutic implications, and have added biological context about cell proliferation/differentiation balance.

The revised paragraph (p. 18-19) now reads (with additions in bold and problematic phrasing removed):

"In conclusion, our findings regarding the pivotal role of ZYG-8^{DCLK1} in spindle positioning and orientation, primarily through rigidifying microtubules during mitosis, offer not only a deeper understanding of the fundamental mechanisms governing cell division but also highlight significant implications for cancer therapy. Given that **accurate spindle positioning is essential for maintaining the balance between cell proliferation and differentiation, our results may have broader relevance for understanding how disruptions in these processes contribute to tumour initiation and progression [170-172]. The frequent deregulation of** DCLK1 in various solid tumours (e.g., those from the colon, pancreas, kidney and breast) suggests its involvement in a common process such as cell division [173-176]. Additionally, the proteins of the Tau family are also found to be deregulated or mutated in cancers [177-179]. **While our study focuses on fundamental mechanisms, these observations raise the possibility that microtubule rigidity could represent an underexplored aspect of cancer cell biology**."

Similarly, we have revised the end of the abstract as follows:

"Given that DCLK1 is frequently deregulated in human cancers and that accurate spindle positioning is essential for maintaining cell proliferation-differentiation balance, these findings may have implications for understanding how disruptions in microtubule mechanics contribute to carcinogenesis."

11. References could to be streamlined with DOI.

We thank the reviewer for this suggestion. We have now updated all references in the manuscript to include their respective DOIs.
