# Supplementary material for: Microtubule stiffening by the doublecortin-domain protein ZYG-8 contributes to mitotic spindle orientation during zygote division in Caenorhabditis elegans
Source: PLoS Genet. 2026 Jun 29;22(6):e1012196. doi: 10.1371/journal.pgen.1012196 (PMC13340844; doi:10.1371/journal.pgen.1012196)
Supplement: Peer review history

# Author comment: Author response to Decision Letter 0

**DOI:** 10.1371/journal.pgen.1012196.r001

**Transfer Alert**

This paper was transferred from another journal. As a result, its full editorial history (including decision letters, peer reviews and author responses) may not be present.

21 Oct 2025

# Peer review: Decision Letter 0

**DOI:** 10.1371/journal.pgen.1012196.r002

**Authors:** Pablo Wappner, Amy E. Ikui

10 Dec 2025

PGENETICS-D-25-01140

Microtubule stiffening by the doublecortin-domain protein ZYG-8 contributes to mitotic spindle orientation during zygote division in Caenorhabditis elegans.

PLOS Genetics

Dear Dr. Bouvrais,

Thank you for submitting your manuscript to PLOS Genetics. After careful consideration, we feel that it has merit but does not fully meet PLOS Genetics's publication criteria as it currently stands. Therefore, we invite you to submit a revised version of the manuscript that addresses the points raised during the review process.

Please submit your revised manuscript by Feb 08 2026 11:59PM. If you will need more time than this to complete your revisions, please reply to this message or contact the journal office at plosgenetics@plos.org. When you're ready to submit your revision, log on to https://www.editorialmanager.com/pgenetics/ and select the 'Submissions Needing Revision' folder to locate your manuscript file.

Please include the following items when submitting your revised manuscript:

* A rebuttal letter that responds to each point raised by the editor and reviewer(s). You should upload this letter as a separate file labeled 'Response to Reviewers'. This file does not need to include responses to any formatting updates and technical items listed in the 'Journal Requirements' section below.

* A marked-up copy of your manuscript that highlights changes made to the original version. You should upload this as a separate file labeled 'Revised Manuscript with Track Changes'.

* An unmarked version of your revised paper without tracked changes. You should upload this as a separate file labeled 'Manuscript'.

If you would like to make changes to your financial disclosure, competing interests statement, or data availability statement, please make these updates within the submission form at the time of resubmission. Guidelines for resubmitting your figure files are available below the reviewer comments at the end of this letter.

We look forward to receiving your revised manuscript.

Kind regards,

Amy E. Ikui

Academic Editor

PLOS Genetics

Pablo Wappner

Section Editor

PLOS Genetics

Aimée Dudley

Editor-in-Chief

PLOS Genetics

Anne Goriely

Editor-in-Chief

PLOS Genetics

**Journal Requirements:**

1) We ask that a manuscript source file is provided at Revision. Please upload your manuscript file as a .doc, .docx, .rtf or .tex. If you are providing a .tex file, please upload it under the item type u2018LaTeX Source Fileu2019 and leave your .pdf version as the item type u2018Manuscriptu2019.

2) Please provide an Author Summary. This should appear in your manuscript between the Abstract (if applicable) and the Introduction, and should be 150-200 words long. The aim should be to make your findings accessible to a wide audience that includes both scientists and non-scientists. Sample summaries can be found on our website under Submission Guidelines:

https://journals.plos.org/plosgenetics/s/submission-guidelines#loc-parts-of-a-submission

3) We do not publish any copyright or trademark symbols that usually accompany proprietary names, eg ©,  ®, or TM  (e.g. next to drug or reagent names). Therefore please remove all instances of trademark/copyright symbols throughout the text, including:

- ® on page: 41.

4) Please upload all main figures as separate Figure files in .tif or .eps format. For more information about how to convert and format your figure files please see our guidelines:

https://journals.plos.org/plosgenetics/s/figures

5) We have noticed that you have uploaded Supporting Information files, but you have not included a list of legends. Please add a full list of legends for your Supporting Information files after the references list.

6) Please amend your detailed Financial Disclosure statement. This is published with the article. It must therefore be completed in full sentences and contain the exact wording you wish to be published.

State what role the funders took in the study. If the funders had no role in your study, please state: "The funders had no role in study design, data collection and analysis, decision to publish, or preparation of the manuscript.".

If you did not receive any funding for this study, please simply state: u201cThe authors received no specific funding for this work.u201d

**Reviewers' comments:**

Reviewer's Responses to Questions

**Comments to the Authors:**

**Please note here if the review is uploaded as an attachment.**

Reviewer #1: Summary

This manuscript by Cueff et al investigates how the doublecortin domain protein ZYG 8 regulates spindle positioning and oscillations in the C. elegans zygote. Using three perturbation strategies, (i) a temperature sensitive (ts) mutant affecting the first doublecortin domain, (ii) overexpression by additional expression of ZYG-8 from the pie 1 promoter, and (iii) RNAi depletion, the authors show that ZYG-8 localizes to the mitotic spindle, including astral microtubules, and that its loss increases the oscillation amplitude while decreasing frequency. They further analyze microtubule (MT) growth and nucleation via EBP-2 tracking and centrosome size, examine cortical MT contact dynamics, and propose that ZYG-8 modulates MT flexural rigidity, thereby altering the balance of pushing and pulling forces on the spindle.

The topic is significant, and the dataset is rich, but the manuscript is dense and sometimes confusing, and several comparisons (e.g., SPD-2, CLS-2) are not the most informative. The rationale for analyzing cortical MT contacts and DiLiPop separately is unclear, as both address MT number and lifetime at the cortex, with the only difference that in the DiLiPop assay the authors classify short MT lifetimes at the cortex as pulling and long life times as pushing events. Overall, the work is interesting and relevant but requires some clarification.

Major Strengths

Multiple independent perturbations (CRISPR ts allele, overexpression, RNAi) increase confidence that phenotypes are due to ZYG-8.

Clear primary phenotype: ZYG-8 loss increases oscillation amplitude and reduces frequency; ts allele shows stronger effect than RNAi, consistent with reduced MT binding.

Integration of approaches: imaging, quantitative analysis, and modeling.

Novel mechanistic model linking doublecortin-dependent MT rigidity to spindle force balance.

Major Concerns

1) The logic excluding growth and nucleation effects is hard to follow because comparisons are not equivalent in magnitude or mechanism.

As an example, the ZYG 8 ts reduces the MT growth rate by ~15%, to exclude that this reduction in growth rate is what affects the oscillation, the authors choose RNAi of the MT polymerase ZYG-9. Depletion of ZYG 9 was reported to reduce MT growth rates by ~3 fold and to shorten spindles. Even though the authors use partial RNAi, which presumably has less effect on MT growth rates, those conditions might not be comparable and variable.

In addition, the authors find that ZYG 8 depletion increases MT nucleation, to exclude an effect of increased MT nucleation on oscillations the authors choose to compare ZYG-8 depletion to SPD-2 depletion. However, SPD 2 depletion decreases MT nucleation, which is the opposite as reported for ZYG-8. Thus, it is not a good control to test the effect on oscillations of an increased MT nucleation. The authors should consider instead a perturbation that elevates nucleation, i.e. KLP-7 (however there are caveats with KLP-7 as well, see below).

The section comparing ZYG 8 to CLASP (CLS 2) and KLP 7 is difficult to interpret because both of these perturbations strongly affect midzone integrity and frequently cause spindle rupture, unlike ZYG 8 manipulations. In addition, both KLP-7 and CLS-2 are mainly acting on the chromosomes to correct errors and promote kinetochore MT growth, respectively. Since both KLP-7 and CLS-2, alter global spindle architecture and do not just affect global MT polymerization, they are not very good mechanistic controls for astral MT behavior. If the aim is to control for polymerase activity, ZYG 9 (carefully titrated) would be the more appropriate control.

Overall, while those comparisons are used by the authors to emphasize that changes in MT dynamics alone do not cause the same effects on oscillations, I am not sure they are helpful in supporting this message. I think these comparisons could be moved to Supplement and it would be helpful to point out the limitations of each of them in the discussion.

2) The rationale for analyzing cortical MT contacts and DiLiPop separately is unclear since both assess MT number and lifetime at the cortex. The mayor difference seems to be that depending on the lifetime (short vs long) the microtubules are counted as pulling or pushing.

Based on the DiLiPop assay the authors discard an effect of pulling, which seems a bit unclear. While they detect longer lifetimes, which the authors argue are indicative of more pushing they also state that the measured reduction in density of pulling events (short time) could be due to prolonged duration of individual pulling. This would however mean that some of the longer time events could then actually be pulling events and not pushing events and would thus be falsely classified by the assay. Maybe the authors could clarify this.

3) Evidence for pulling forces being “largely unaffected” relies on indirect metrics (centrosome position, DiLiPop) and is partially contradictory.

The authors could consider laser ablation to directly test pulling forces, or acknowledge limitations.

Along this line, On Page 19: posterior centrosome position is used to argue that there is no change in pulling forces as the position is comparable to wildtyp after zyg-8 (RNAi), in contrast page 22 states that in the ZYG 8 ts, the centrosome is closer to the cortex. Please reconcile this difference.

Cytosim simulations are a strength, but parameter choices (rigidity values from in vitro data) should be justified and ideally linked to measured curvature distributions.

4) The figures could benefit from more clear organization and consistency. Along this line, the figures sometimes show only RNAi or only ts mutant, not both. This can appear selective.

As an example, Figure 4 F shows the relative contact count for zyg-8 RNAi for contacts between 0.2-0.3s and 0.3-0.4s, the data the reader should compare this to is in Figure 4H. However, Figure 4H shows the relative count for zyg-8 overexpression and only for events longer than 0.4s, this makes it difficult to compare as the two plots show essentially data for different groups of MT contacts. Data for the ZYG-8 ts strain is not shown at all in Figure 4, while it appeared in previous figures.

Similarly Figure 5 only shows data for ZYG-8 ts and control but not for zyg-8 (RNAi) or overexpression. Figure 5 could also be combined with Figure 6.

In Figure 6 A and B the data shows the curvature for ZYG-8 (RNAi) but then C and D show the proportion of MTs with a certain curvature for the ZYG-8 ts mutant.

In Figure 7 only data for the DiLiPop assay in zyg-8 (RNAi) is shown, no data for the ZYG-8 ts or overexpression.

Figure 8 shows only data for the TS mutant.

The use of different conditions in different figures should either be justified, i.e. why does Figure 7 only show data for zyg-8 (RNAi) and Figure 8 only the TS mutant, or the different conditions should be consistently included in main figures.

Typo: Fig. 7B, in the text it says the figure shows pushing but based on the figure I believe it should be pulling.

In summary, the core findings are interesting and potentially impactful. Addressing the comparability of controls, clarifying analyses, and resolving text inconsistencies will substantially improve the manuscript.

Reviewer #2: The manuscript by Cueff et al. focuses on the role of ZYG8/DCX in spindle orientation in C. elegans. The manuscript's strength lies in the number and variety of microscopy-based quantifications performed on fluorescently labeled microtubule data from both live and fixed samples. The authors identify spindle phenotypes associated with ZYG8 perturbations and explore multiple avenues to elucidate the underlying mechanism explaining these phenotypes. Authors demonstrate that ZYG8 perturbations result in a reduction in microtubule growth rate (Figure 2), an increase in microtubule nucleation (Figure 3), and an increase in cortical contacts (Figure 4). Can these subcellular microtubule-associated phenotypes explain the spindle oscillation phenotype? To answer this question, the authors compare the effect of ZYG8 perturbations on spindle oscillations with that of other genes involved in regulating microtubule growth rate (XMAP), nucleation (CEP192), stabilization (CLASP), destabilization (MCAK), or stiffness (TAU). All these explorations lead the authors to propose that the role of ZYG8 in spindle orientation and oscillations stems not from its involvement in microtubule dynamics, but rather from its effect on microtubule stiffness. I found that the quantification and analysis of ZYG8 phenotypes were well done and convincing; however, the argument regarding microtubule rigidity as the unique causal parameter was somewhat weaker. Indeed, the microtubule shape analysis used to infer microtubule stiffness is based on an untested assumption, and the comparisons between ZYG8 and other microtubule regulators are incomplete, as microtubule properties (dynamics, rigidity) are not systematically analyzed in the conditions where known microtubule regulators are perturbed, only spindle phenotypes are. Overall, the strengths of the manuscript provide a solid foundation on which the authors can further refine their mechanistic conclusions. Below are some suggestions that I hope will be helpful in strengthening the manuscript's main points.

Major comments:

• Measuring microtubule stiffness or rigidity is very difficult, if not impossible, in cells, as recognized by the authors at the beginning of section 3 of the results. The authors should be commended for tackling an inherently challenging question and for being transparent about the limitations. The authors decide to rely on a shape analysis to deduce potential changes in rigidity. This is a very classical comparison often made in mechanics classes: if two beams are subjected to the same load, but one bends more than the other, then their mechanical properties must be different. The converse is equally true: if two beams have different shapes under load but have the same mechanical properties, the loads must be different. One difference is that in the first case, where identical loads but different mechanical properties are involved, the external forces are the same for both beams—they push back on the load with the same overall force— even though they bend by different amounts.

If we let the beams in our examples be microtubules, the thought experiment remains valid. However, there are now two unknown parameters, the load and the mechanical properties, which prevent reaching a conclusion just from the shape analysis alone. Again, the authors recognize that limitation and decide to work “under the assumption that the forces acting on the microtubule network are similar between conditions.” This assumption is critical, as it determines how strongly the conclusions can be supported. Because the assumption is so critical to the reasoning, and because authors are making rigidity a big part of their story, it is crucial to test the validity of this assumption. I appreciate that the authors are thinking carefully about force-generating mechanisms as demonstrated by the initial analysis provided.

The astral microtubules can experience force from three different sources: dynein motors anchored to the cortex, dynein motors moving cargo along the microtubules, and cortical pushing when the microtubules grow against the cell cortex. The authors must estimate how each of these contributors to forces is affected by the perturbations in ZYG8 to determine if the assumption is valid. I recognize that they partially do it by measuring the number and duration of the cortical contacts. However, while these data begin to address the question, additional information would clarify the robustness the assumption.

The amount of dynein could change, the amount of dynein engaged at the cortex, or on organelles could change, the distribution of organelles could change, the amount of dynein per microtubule could change, etc... The effect of ZYG8 perturbations on the amount of force originating from dynein activity and localization needs to be looked at to properly assess the validity of the assumption and, consequently this part of the reasoning. If additional supporting data are not available, it may help to moderate the strength of the conclusion about rigidity. Critically, a paragraph in section 4 of the results seems to suggest that astral microtubules in CTRL and ZYG8 backgrounds are not experiencing the same force: “We interpreted the decrease in fc as primarily reflecting a centering force reduction due to softer microtubules.” which would indicate that the assumption is not true.

• When authors discuss the dynamics of cortical microtubules (section 2 of the Results), they first describe the number of contacts and their duration in various ZYG8 perturbations. The idea here is, on the one hand, to understand if ZYG8 is a microtubule stabilizer (like other DCX family members) and if that role could explain the phenotypes observed on the spindle. The data indicate that ZYG8 is not a stabilizer in their hands but the opposite. Then, to try to tease out whether the destabilizing role of ZYG8 on microtubules can explain the spindle phenotypes, the authors compare ZYG8 conditions to those of two mutants, CLASP and MCAK. That comparison remains superficial, as the authors only examined spindle orientation in these mutants. Extending the authors’ thoughtful comparison to include additional microtubule-level analyses would further strengthen their argument. To fully understand the picture, authors should analyze the dynamics of cortical microtubules and microtubule shape distribution in these mutants in the same way they did for the ZYG8 conditions (number of contacts and duration, tortuosity, and curvature). It would first validate that CLASP and MCAK are indeed acting as stabilizers and destabilizers, respectively, and importantly, that their analysis pipeline can detect it, supporting the ZYG8 analysis. Notably, since the CLASP mutant exhibits some spindle phenotypes (albeit different from those of ZYG8), it would be interesting to understand if the dynamics of cortical microtubules are altered or if microtubule shape profile is changed in this background, and whether this can explain the observed spindle phenotypes.

• To assess if microtubule rigidity can explain the phenotypes observed authors decided to explore the phenotype associated with PTL-1 depletion as a positive control. I value and support this attempt, but it seems incomplete on two fronts. Firstly, the authors only looked at spindle orientation phenotypes. Comparing the cortical microtubule dynamics and microtubule shape associated with PTL-1 RNAi with those of ZYG8 perturbations would be a more effective way to assess the similarities in mechanism between the two conditions. Secondly, another microtubule rigidity regulator exists and appears to be expressed in C. elegans, SPD-1, which belongs to the Ase1/PRC1/MAP65-1 family. Exploring the spindle orientation phenotypes and cortical microtubule dynamics phenotypes in SPD-1 depletion seems important. Demonstrating that the depletion of three independent regulators, two of which are known to regulate microtubule rigidity, produces similar phenotypes is the most effective way to convince readers that altering microtubule rigidity causes spindle orientation defects independently of the effects on microtubule dynamics in the third regulator, ZYG-8.

In summary, for my major concerns, the authors should look at potential changes in dynein activity and/or localization in ZYG8 perturbation to validate the “equal forces” assumption and complete the datasets on CLASP, MCAK, XMAP, and TAU with microtubule-associated phenotypes (cortical dynamics and shape analysis) to better discriminate which microtubule-level phenotypes are associated with spindle-level phenotypes. If the authors cannot provide all these new datasets, it should not prevent the publication of the manuscript, but the text would need to be amended to soften the conclusions regarding rigidity and discuss the limitations of the provided datasets.

Minor comments:

The manuscript is generally clearly written, and addressing the points below will further improve clarity and reproducibility

• The figure legends, and materials and methods sections do not specify the number of replicates.

In figure 1 for example “We tracked the centrosomes and analysed their positions in: (red) N = 16 zyg-8(RNAi)-treated embryos and (black) N = 10 control RNAi embryos expressing GFP::TBB-2; (light green) N = 21 zyg-8(or484ts) mutants and (light blue) N = 17 untreated embryos, both at the restrictive temperature (Rest. T°) expressing GFP::TBB-2; (purple) N = 19 zyg-8 overexpressing embryos and (grey) N = 17 untreated embryos expressing mCherry::tubulin (Method M7).” Is the number of embryos specified here coming from a single experimental replicate or more? This problem is present throughout the manuscript in many, if not all, of the figures.

The authors should clarify the number of experimental replicates and indicate whether they consider it an appropriate number of animals analyzed per replicate, given the statistical analysis they are conducting.

Similarly, for figures where authors measured subcellular objects that can be present in multiple copies per cell, such as microtubule comets, they should specify not only the number of objects but also the number of embryos from which the data are derived. For example, in the legend of figure 2 it says:” Growth rates of astral microtubules measured at (cross) anterior and (circle) posterior centrosomes: (B) (red) N = 27 comets from anterior centrosome and N = 23 comets from posterior centrosome in 10 zyg-8(RNAi)-treated embryos, and (black) N = 26 comets from anterior centrosome and N = 24 comets from posterior centrosome in 8 control RNAi embryos;” I can imagine that one single spindle could contain hundreds or thousands of growth events from which a subset will be satisfactory for analysis. So, the numbers of comets here seem small to me. Is this number of comets coming from one single embryo or from the aggregate of multiple experiments? How many comets are analyzed per spindle, then, and is it appropriate?

This issue is present in multiple figures, notably Figures 2, 4, and 6. For these figures (and all others where it is appropriate), the authors should indicate the number of animals used per replicate, the number of objects analyzed per replicate, and the number of replicates.

• Continuing with the topic of my previous comment, I think authors should strongly consider representing the data as SuperPlots (https://doi.org/10.1083/jcb.202001064). This will greatly help the reader evaluate the spread in the data, the number of replicates used, and the number of individuals per replicate, allowing them to easily assess the appropriateness of the experimental design. I am thinking about Figure 1 C and D, Figure 2 B, C, and D, Figure 3 B, C, D, G, and H, Figure 4 F and H, Figure 6 E, F, G, and H, and Figure 8 C and D.

• For the microtubule shape analysis, it would be a great help to the reader if the authors could provide a side-by-side of the spindle image and the resulting traces. The authors provide only a single side-by-side example in Figure S8, without indicating whether this represents a CTRL or perturbed embryo. I would suggest providing a few side-by-sides for each condition analyzed. This will showcase the methodology and allow readers to perform a visual validation.

• “A previous study showed that microtubules in axonal growth cones were more often strongly curved upon depletion of the Doublecortin-family proteins DCX and DCLK1.” This statement should be supported by a reference.

• “Figure 7: Depleting ZYG-8 weakens highly the cortical pushing events.” This figure title needs correcting for its English.

• Considering SPD-1/Ase1/PRC1/MAP65-1, the text states “Research, particularly in neuronal systems, has identified two major protein families involved in regulating microtubule rigidity […] the Doublecortin family, […] and the MAP2/MAP4/Tau superfamily.” This statement overlooks the fact that the SPD-1/Ase1/PRC1/MAP65-1 family is involved in microtubule rigidity, albeit not in neuronal systems, and has been proposed to affect microtubule rigidity independently of microtubule dynamics. See https://doi.org/10.1091/mbc.e13-03-0141 or https://doi.org/10.7554/eLife.53807, for example. The statement, or the paragraph, could mention this family of proteins.

Reviewer #3: Cueff et al., 2025 report a microtubule stiffening role for ZYG-8 in regulating mitotic spindle orientation. Using 3 complementary methods, the authors disrupt the fuction of zyg-8/DCLK1, the C. elegans Doublecortin and find incorrect mitotic spindle positioning, as reported earlier. In the zygote, ZYG-8 modestly promotes microtubule growth and limits nucleation. Further analysis showed changes in spindle pole oscillations and microtubule cortical-contact behaviour in Zyg-8 depleted cells. ZYG-8 depletion or mutation led to curved microtubules and increased cortical life-time. Using simulations, the authors show that that reduced rigidity can increase cortical lifetimes. They correlate microtubule softening in zyg-8 depleted embryos and mutants with decreased spindle centering, which may be linked to increased spindle oscillations. During anaphase the authors find spindle poles travelling closer to cell periphery associated with mispositioned spindles. Overall, most observations have high quality images, although the display of measurements (graphs) can be strengthened. While its possible that microtubule curvature may play a significant role in regulating Zyg8 disruption induced spindle positioning phenotype, the evidence for ruling out microtubule dynamics is somewhat weak. This is at least in part because it’s difficult to rigorously correlate spindle movements with 3D growth rates of microtubules - at multiple regions of the spindle - through time. The authors could manage this knowledge gap with a balanced discussion rather than omitting the role of microtubule dynamics altogether.

In general, I find this paper very strong with high-resolution movies; some graphs and discussion can be strengthened.

Major points:

1) Cueff et al., indicate “DCX proteins bind to microtubules and are thought to stabilise or rigidify them.” Doublecortin in human cells induces mitotic microtubule catastrophe – they show its relevance to glioblastoma (Santra J Neruochem 2009). It will help readers if the authors discuss the past DCX studies in the context of new findings. For example, what is the relationship between microtubule stiffening and microtubule dynamics (or catastrophe). This is particularly important as the authors propose implications for cancer therapy, by stating doublecortin is deregulated in human cancer.

2. Abstract rules out microtubule growth defects as a reason for change in oscillations. But Discussion section indicates “… a dual role for ZYG-8DCLK1 during mitosis, involving the control of both microtubule rigidity and dynamics.” Ruling out of microtubule dynamics in spindle movements appears to be based on indirect outcomes. For example, through comparing ZYG-9 partial depletion that achieves a partial reduction in microtubule growth rate: “16% reduction in microtubule growth rate, comparable to that observed in zyg-8 mutants (Figure S4B).”. This type of indirect comparison does not rule out the possibility of 15% change in microtubule growth in zyg-8 cells have a role in addition to changes in microtubule rigidity. This is important because disruption of PTL-1/Tau known to reduce microtubule rigidity causes milder centring defects compared to zyg-9 loss.

3. Impaired pushing forces have been reported following Mark2/Par1b loss (see below): Excessive defects with spindle-centring and spindle oscillations have been observed in human cells lacking MARK2/Par1 kinase (Zulkipli et al., 2018 Journal of Cell Biology) or following MARK2 inhibitor treatment (Dang et al., 2023 Journal of Cell Biology). They have been reported to be independent of cortical dynein. These published works should be either introduced in introduction, or at least considered in discussion.

4. Figure 5 images show a clear difference in microtubule structure, but Figure 6 curvature measurements need statistical significance. Curvature measurement method can help reader. I would suggest merging these two figures so that it easier to see the graphs on the side of the images.

6. Figure 6: sample sizes are somewhat low. Particularly 6A has only 5 samples.

5. In Figure 7a – the model, the three sections of the cartoon is somewhat unclear: in section 3,why would the rigid microtubule that started depolymerising in section 2 continue to be in depolymerising state (This microtubule marked in green could be reversing to growth phase or remaining as such as there is no cortical contact push after the initiation of depolymerisation in stage 2).

6. The authors propose in abstract “..sufficient microtubule rigidity is essential for generating effective cortical pushing forces, which contribute to centring mechanisms...”

I agree with the author’s statement in discussion that it is “important to explore the mechanisms by which ZYG-8 rigidifies the microtubules.” However, the sentence in abstract quoted above is built on several correlative evidence, or lack of severe phenotypes, by studying different types of depletions and mutant conditions.

I would recommend a cautious model that does not exclude any of the moderate phenotypes, particularly those that are not directly measured (for example, microtubule catastrophe rates) or not highly striking (for example, changes to microtubule growth).

Minor points

1. Please explain biophysical assays in results sections. Reference to such assays are found in abstract and introduction but not within results.

2. Unclear how microtubule growth rates were observed (was it between 10 consecutive time points for comets moving in XY or XYZ .

3. Please explain why it needs to be measured 50 times: “We repeated the measurement approximately 50 times for each condiHon across about ten embryos (about 25 comets per centrosome).”

4. Figure 3E needs scale bars.

5. Are the spindle mispositioning phenotypes most prominent in anaphase? Would a microtubule curvature induced phenotype reduce with reducing microtubule length?

6. Would microtubules experience reduced tortuosity as they are being pulled compared to those that are not being pulled by cortical dynein?

7. The observations does not rule out asymmetric regulation of cortex reaching microtubules from one pole but not the other “Thirdly, we show that in zyg-8(or484ts) mutants, astral microtubules are still able to contact the opposite cortex (Figure 5A, Movies S5 and S6). This rules out a model in which spindle misorientation arises from a failure of microtubules to reach the cortex…,”.

8. Figure 8 panel B show prominent astral microtubules on the anterior spindle – please comment.

9. Include Zulkipli et al., 2018 while discussing metaphase only phenotypes here:

“To date, cortical pushing forces have been proposed to contribute to spindle centring during metaphase [25, 27].”

10. These discussion sentences in quotes below are somewhat unclear and feel stretched considering the protein is involved in embryonic development and cell division – the discussion is strong without these extended therapeutic plans. “Given that DCLK1 is frequently deregulated in various solid tumours (e.g., those from the colon, pancreas, kidney and breast), this suggests its involvement in a common process such as cell division [154-157]. Additionally, as the proteins of the Tau family are also found to be deregulated or mutated in cancers, targeting microtubule rigidity may represent a promising approach for future cancer treatments [158-160]. This strategy could potentialise existing therapies targeting microtubule dynamics, which ooen suffer from side effects and are limited by cancer cell resistance.”

11. References could to be streamlined with DOI.

**********

**Have all data underlying the figures and results presented in the manuscript been provided?**

Large-scale datasets should be made available via a public repository as described in the *PLOS Genetics*  data availability policy, and numerical data that underlies graphs or summary statistics should be provided in spreadsheet form as supporting information.

Reviewer #1: Yes

Reviewer #2: Yes

Reviewer #3: Yes

**********

PLOS authors have the option to publish the peer review history of their article (what does this mean?). If published, this will include your full peer review and any attached files.

If you choose “no”, your identity will remain anonymous but your review may still be made public.

**Do you want your identity to be public for this peer review?**  For information about this choice, including consent withdrawal, please see our Privacy Policy.

Reviewer #1: No

Reviewer #2: No

Reviewer #3: No

[NOTE: If reviewer comments were submitted as an attachment file, they will be attached to this email and accessible via the submission site. Please log into your account, locate the manuscript record, and check for the action link "View Attachments". If this link does not appear, there are no attachment files.]

**Figure resubmission:**

While revising your submission, we strongly recommend that you use PLOS’s NAAS tool (https://ngplosjournals.pagemajik.ai/artanalysis) to test your figure files. NAAS can convert your figure files to the TIFF file type and meet basic requirements (such as print size, resolution), or provide you with a report on issues that do not meet our requirements and that NAAS cannot fix.

After uploading your figures to PLOS’s NAAS tool - https://ngplosjournals.pagemajik.ai/artanalysis, NAAS will process the files provided and display the results in the "Uploaded Files" section of the page as the processing is complete. If the uploaded figures meet our requirements (or NAAS is able to fix the files to meet our requirements), the figure will be marked as "fixed" above. If NAAS is unable to fix the files, a red "failed" label will appear above. When NAAS has confirmed that the figure files meet our requirements, please download the file via the download option, and include these NAAS processed figure files when submitting your revised manuscript.

**Reproducibility:**

To enhance the reproducibility of your results, we recommend that authors of applicable studies deposit laboratory protocols in protocols.io, where a protocol can be assigned its own identifier (DOI) such that it can be cited independently in the future. Additionally, PLOS ONE offers an option to publish peer-reviewed clinical study protocols. Read more information on sharing protocols at https://plos.org/protocols?utm_medium=editorial-email&utm_source=authorletters&utm_campaign=protocols

# Author comment: Author response to Decision Letter 1

**DOI:** 10.1371/journal.pgen.1012196.r003

21 Apr 2026

Attachment Submitted filename: Cueff_2026_Response_to_reviewers.pdf

# Peer review: Decision Letter 1

**DOI:** 10.1371/journal.pgen.1012196.r004

**Authors:** Pablo Wappner, Amy E. Ikui

1 Jun 2026

Dear Dr Bouvrais,

We are pleased to inform you that your manuscript entitled "Microtubule stiffening by the doublecortin-domain protein ZYG-8 contributes to mitotic spindle orientation during zygote division in Caenorhabditis elegans." has been editorially accepted for publication in PLOS Genetics. Congratulations!

Before your submission can be formally accepted and sent to production you will need to complete our formatting changes, which you will receive in a follow up email. Please be aware that it may take several days for you to receive this email; during this time no action is required by you. Please note: the accept date on your published article will reflect the date of this provisional acceptance, but your manuscript will not be scheduled for publication until the required changes have been made.

Once your paper is formally accepted, an uncorrected proof of your manuscript will be published online ahead of the final version, unless you’ve already opted out via the online submission form. If, for any reason, you do not want an earlier version of your manuscript published online or are unsure if you have already indicated as such, please let the journal staff know immediately at plosgenetics@plos.org.

In the meantime, please log into Editorial Manager at https://www.editorialmanager.com/pgenetics/, click the "Update My Information" link at the top of the page, and update your user information to ensure an efficient production and billing process. Note that PLOS requires an ORCID iD for all corresponding authors. Therefore, please ensure that you have an ORCID iD and that it is validated in Editorial Manager. To do this, go to ‘Update my Information’ (in the upper left-hand corner of the main menu), and click on the Fetch/Validate link next to the ORCID field.  This will take you to the ORCID site and allow you to create a new iD or authenticate a pre-existing iD in Editorial Manager.

If you have a press-related query, or would like to know about making your underlying data available (as you will be aware, this is required for publication), please see the end of this email. If your institution or institutions have a press office, please notify them about your upcoming article at this point, to enable them to help maximise its impact. Inform journal staff as soon as possible if you are preparing a press release for your article and need a publication date.

Thank you again for supporting open-access publishing; we are looking forward to publishing your work in PLOS Genetics!

Yours sincerely,

Amy E. Ikui

Academic Editor

PLOS Genetics

Pablo Wappner

Section Editor

PLOS Genetics

Aimée Dudley

Editor-in-Chief

PLOS Genetics

Anne Goriely

Editor-in-Chief

PLOS Genetics

www.plosgenetics.org

BlueSky: @plos.bsky.social

----------------------------------------------------

Comments from the reviewers (if applicable):

Both reviewers agreed that the authors have addressed all of their concerns following careful revision. The manuscript has been substantially improved and now meets the standard expected for the data presentation and conclusion.

Reviewer's Responses to Questions

**Comments to the Authors:**

**Please note here if the review is uploaded as an attachment.**

Reviewer #1: I thank the authors for their thorough and careful revision. They have fully addressed my major concerns. The addition of the gbp-1(RNAi) comparison in the DiLiPop analysis (new Figure 7F–I) is a good and convincing experiment that strengthens the conclusion that cortical pulling forces are not substantially increased upon zyg-8(RNAi). The new cortical dynein tracking data further support this interpretation. The clarification of the SPD-2 control rationale and the revised ZYG-9 titration logic are now much clearer and I am content with these explanations. The reconciliation of the two centrosome position measurements is also appreciated. Regarding figure consistency, I agree with the authors' justification for keeping the KLP-7 and CLS-2 comparisons in the main text, and the addition of a dedicated discussion paragraph acknowledging their limitations addresses my concern. The typo in Figure 7B has been corrected. In summary, I am satisfied with the revision and support publication.

Reviewer #3: The authors have successfully addressed all my comments, improved their figures and strengthened the article further through extensive revision.

**********

**Have all data underlying the figures and results presented in the manuscript been provided?**

Large-scale datasets should be made available via a public repository as described in the *PLOS Genetics*  data availability policy, and numerical data that underlies graphs or summary statistics should be provided in spreadsheet form as supporting information.

Reviewer #1: Yes

Reviewer #3: Yes

**********

PLOS authors have the option to publish the peer review history of their article (what does this mean?). If published, this will include your full peer review and any attached files.

If you choose “no”, your identity will remain anonymous but your review may still be made public.

**Do you want your identity to be public for this peer review?**  For information about this choice, including consent withdrawal, please see our Privacy Policy.

Reviewer #1: No

Reviewer #3: No

----------------------------------------------------

**Data Deposition**

If you have submitted a Research Article or Front Matter that has associated data that are not suitable for deposition in a subject-specific public repository (such as GenBank or ArrayExpress), one way to make that data available is to deposit it in the Dryad Digital Repository. As you may recall, we ask all authors to agree to make data available; this is one way to achieve that. A full list of recommended repositories can be found on our website.

The following link will take you to the Dryad record for your article, so you won't have to re‐enter its bibliographic information, and can upload your files directly:

http://datadryad.org/submit?journalID=pgenetics&manu=PGENETICS-D-25-01140R1

More information about depositing data in Dryad is available at http://www.datadryad.org/depositing. If you experience any difficulties in submitting your data, please contact help@datadryad.org for support.

Additionally, please be aware that our data availability policy requires that all numerical data underlying display items are included with the submission, and you will need to provide this before we can formally accept your manuscript, if not already present.

----------------------------------------------------

**Press Queries**

If you or your institution will be preparing press materials for this manuscript, or if you need to know your paper's publication date for media purposes, please inform the journal staff as soon as possible so that your submission can be scheduled accordingly. Your manuscript will remain under a strict press embargo until the publication date and time. This means an early version of your manuscript will not be published ahead of your final version. PLOS Genetics may also choose to issue a press release for your article. If there's anything the journal should know or you'd like more information, please get in touch via plosgenetics@plos.org.

# Editor report: Acceptance letter

**DOI:** 10.1371/journal.pgen.1012196.r005

**Authors:** Pablo Wappner, Amy E. Ikui

PGENETICS-D-25-01140R1

Microtubule stiffening by the doublecortin-domain protein ZYG-8 contributes to mitotic spindle orientation during zygote division in Caenorhabditis elegans.

Dear Dr Bouvrais,

We are pleased to inform you that your manuscript entitled "

Microtubule stiffening by the doublecortin-domain protein ZYG-8 contributes to mitotic spindle orientation during zygote division in Caenorhabditis elegans." has been formally accepted for publication in PLOS Genetics! Your manuscript is now with our production department and you will be notified of the publication date in due course.

The corresponding author will soon be receiving a typeset proof for review, to ensure errors have not been introduced during production. Please review the PDF proof of your manuscript carefully, as this is the last chance to correct any errors. Please note that major changes, or those which affect the scientific understanding of the work, will likely cause delays to the publication date of your manuscript.

Soon after your final files are uploaded, unless you have opted out or your manuscript is a front-matter piece, the early version of your manuscript will be published online. The date of the early version will be your article's publication date. The final article will be published to the same URL, and all versions of the paper will be accessible to readers.

For Research Articles, you will receive an invoice from PLOS for your publication fee after your manuscript has reached the completed accept phase. If you receive an email requesting payment before acceptance or for any other service, this may be a phishing scheme. Learn how to identify phishing emails and protect your accounts at https://explore.plos.org/phishing.

Thank you again for supporting PLOS Genetics and open-access publishing. We are looking forward to publishing your work!

With kind regards,

Anitha Samidurai

PLOS Genetics

On behalf of:

The PLOS Genetics Team

Carlyle House, Carlyle Road, Cambridge CB4 3DN | United Kingdom

plosgenetics@plos.org | +44 (0) 1223-442823

plosgenetics.org | Twitter: @PLOSGenetics
